# Identifying patients at risk of unplanned re-hospitalisation using statewide electronic health records

**DOI:** 10.1038/s41598-022-20907-z

**Published:** 2022-10-05

**Authors:** Aida Brankovic, David Rolls, Justin Boyle, Philippa Niven, Sankalp Khanna

**Affiliations:** 1 CSIRO, The Australian e-Health Research Centre, Brisbane, 4029 Australia; 2 CSIRO, The Australian e-Health Research Centre, Parkville, 3052 Australia

**Keywords:** Health care, Health services, Public health, Mathematics and computing, Computational science

## Abstract

Preventing unplanned hospitalisations, including readmissions and re-presentations to the emergency department, is an important strategy for addressing the growing demand for hospital care. Significant successes have been reported from interventions put in place by hospitals to reduce their incidence. However, there is limited use of data-driven algorithms in hospital services to identify patients for enrolment into these intervention programs. Here we present the results of a study aiming to develop algorithms deployable at scale as part of a state government’s initiative to address rehospitalizations and which fills several gaps identified in the state-of-the-art literature. To the best of our knowledge, our study involves the largest-ever sample size for developing risk models. Logistic regression, random forests and gradient boosted techniques were explored as model candidates and validated retrospectively on five years of data from 27 hospitals in Queensland, Australia. The models used a range of predictor variables sourced from state-wide Emergency Department(ED), inpatient, hospital-dispensed medications and hospital-requested pathology databases. The investigation leads to several findings: (i) the advantage of looking at a longer patient data history, (ii) ED and inpatient datasets alone can provide useful information for predicting hospitalisation risk and the addition of medications and pathology test results leads to trivial performance improvements, (iii) predicting readmissions to the hospital was slightly easier than predicting re-presentations to ED after an inpatient stay, which was slightly easier again than predicting re-presentations to ED after an EDstay, (*iv*) a gradient boosted approach (XGBoost) was systematically the most powerful modelling approach across various tests.

## Introduction

An important performance indicator for many health jurisdictions is the return of a patient to hospital shortly after discharge. Such returns threaten the quality of patient care and lead to increased medical care costs^1^. Hospital readmissions cause a disruption to patients’ lives, result in a significant financial burden on the healthcare system and, in many countries, hospitals with high readmission rates are subject to financial penalties. The imperatives of improving quality of patient care and reducing cost has motivated healthcare facilities to reduce their readmission rates by predicting patients who are at high risk of readmission^1–5^. Readmission risk assessment can be used to help target the delivery of interventions to patients at greatest risk^6^. Ideally, models designed for this purpose provide clinically relevant stratification of readmission risk and give information early enough during the hospitalisation to trigger a transitional care intervention, many of which involve discharge planning and begin well before hospital discharge^7^.

The accuracy and reliability of risk models largely depends on the predictors included and methods of development, validation, calibration, and clinical utility^2^. With recent investments in electronic health records (EHR) and their increasing use and application in healthcare systems, readmission risk prediction using EHR has also expanded^2,8,9^. The past few years has seen a surge in the development of highly sophisticated predictive models. In the last decade there have been at least a dozen published systematic reviews of predictive models of readmission^1,2,7,8,10–17^, half of which were published in the last 2 years^1,2,10,13,15–17^.

These systematic reviews reinforce favorable attributes of readmission risk models intended for clinical use: (i) they should have good discrimination (discriminate high- from low-risk patients); (ii) provide current risk scores before a patient is discharged from hospital; (iii) be deployable at scale; (iv) use reliable data that can be easily obtained; and (v) use variables that are clinically related to, and validated in the populations in which use is intended^7^. Yu et al.^18^ recognised the plethora of literature that deals with developing readmission risk prediction models, but noted that most of them do not have sufficient prediction accuracy to be deployed in a clinical setting, partly because different hospitals may have different characteristics in their patient populations.

In addition to the obvious importance of the predictive accuracy of risk models, a serious concern about model evaluation practices has been raised where measures of discrimination (e.g. area under the receiver operating characteristic curve (ROC-AUC)) are emphasised much more than measures of calibration, and when measures of calibration are reported, they are most often reported in a way that is clinically uninterpretable^19^. Model calibration is frequently less stable than discrimination, yet highly consequential to decision making^20^. Stable discrimination relies on the consistency of the effects of the measured variables and the casemix of the population, whereas stable calibration is much more sensitive to effects of unmeasured covariates. The critical importance of poor calibration is frequently underappreciated: poor calibration can lead to harmful decisions^19^. Another aspect typically neglected is the importance of assessing the precision and recall performance of the models. ROC-AUC is threshold independent, and describe prediction performance over the entire operating range, which includes regions of no practical relevance such as the extreme right (high false positive rate) or extreme left side (low true positive rate)^21^. Although ROC is widely used, the Precision-Recall curve (PRC) is an equally important performance metric in that is emphasizes the performance for true positives, which arguably are more important in the problem context. Hence, for the sake of completeness we provide PRC curves in addition to ROC curves for the final models.

Current approaches often distinguish between (and frequently compare) machine learning (ML) and traditional statistical modelling approaches. While some studies have shown ML approaches offer better performance^15,17^, others have reported no significant improvement over traditional regression-based approaches^12^. We argue that both approaches offer strengths and weaknesses. The traditional regression-based approach offers explainability and ease of implementation but does not always provide the highest discrimination and nonlinearities must be specifically specified. With ML, most methods have intrinsic mechanisms to include nonlinearities and most methods can handle collinearities in predictors, but implementation requires more libraries and functions and they do not always provide the highest discrimination. Also, many commonly-used methods are not intrinsically explainable. Hence, we believe that a robust modeling approach should include both approaches.

We also argue that transparency and explainability are an absolute necessity for the widespread introduction of risk models into clinical practice, and models used for clinical decision support must incorporate explainability of individual predictions. Despite a large body of literature, readmission risk prediction remains a poorly understood and complex endeavour^7^ and many studies do not investigate which factors influence a model’s performance or predictions. Being accountable for treatment mistakes, clinicians must be able to understand the underlying reasoning of models so they can trust the predictions^22^. A useful explanation involves the ability to account for the relevant parts in a model leading to a prediction, and also the ability to present this relevance in a comprehensible way. An explanation that is too hard to interpret and comprehend will most likely not have any practical effect^22^.

The primary objective of this study was to develop algorithms deployable in public hospitals across the state of Queensland, Australia, as part of the state government’s initiative to reduce unplanned hospitalisations contributing to the evidence of risk stratification algorithms deployed at scale across large geographic areas. An equally important objective was to develop models that filled gaps in the prior attempts as discussed above. These gaps include developing models with good discrimination and calibration (as recommended by TRIPOD^23^), using a large sample size covering a range of hospitals and hospital sizes, ensuring the factors contributing to an individual’s risk are explainable and understandable, including precision and recall in addition to AUC, and identifying the relative contributions of underlying data collections (comprising ED and inpatient hospital episodes, patient medication information and pathology test results). The developed models can assist hospital and health services in preventing unplanned patient returns to hospital, improving equity in health care access and facilitating individual-based health care planning through the provision of risk scores to treating practitioners.

## Results

### Data characteristics

#### Demographic characteristics

A detailed demographic summary for RP30, RA30 and RP30E outcome metrics is provided in Supplementary information, Tables S17–S19. The demographic summary shows nearly equal gender distribution in all three outcome metrics. Average age was 50 years for re-presentation (RP30) and readmission (RA30) and 40 years for an ED re-presentation (RP30E).

#### Distribution of the outcome metrics

**Figure 1:**
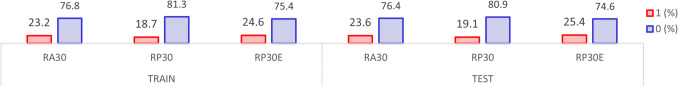
distribution per outcome variable on training and test data. Binary outcome 1 denotes identified admission, presentation and ED presentation occurred within 30 days for RA30, RP30 and RP30E respectively.

Class distribution for each outcome metric in the training and test partition is shown in Fig. 1. As shown, 1 in 4 patients were readmitted or had an ED re-presentation, and 1 in 5 patients had an ED re-presentation to hospital after an inpatient stay within 30 days.

#### Feature counts used for modeling across the cohorts

The total number of features used for modelling was the same across the considered cohorts for the RP30 and RA30 outcome metrics for all data groups. For the RP30 and RA30 outcome metrics, Principal referral and Acute hospitals had the same count of predictors for each data group unlike the Children’s hospital cohort. For the Children’s hospital cohort it ranged from 46 for the ‘Basic’ model to 168 for the ‘All-inclusive DRG’ model. For Principal referral and Acute hospitals it ranged from 90 to 212 for the ‘Basic’ and ‘All-inclusive DRG’ models, respectively. For RP30E, the count of features was the same across all three cohorts and ranged from 30 for the ‘Basic’ model to 117 features for the ‘All-inclusive DRG’ model. Details are omitted for brevity and are available in Supplementary information, Table S12. All details of predictors used in this study and their implementation is provided in Supplementary information Appendix B and C.

### Performance

#### AUC performances of the best and the final models

The best AUC values across the cohorts and outcome metrics obtained for different data groups with Logistic regression (LR) with l1 regularization factor (L1), Random Forest (RF) and Gradient Boosting (XGB) models and the historical window of 180 days are reported in Table 1. Systematically, the best performance was obtained from the XGB approach using the data group ‘All-inclusive DRG model’ followed by XGB trained on ‘Patho’ and ‘All’ data groups while the ‘Expert’ model had the worst performance. AUC performance, the corresponding 95%CIs and the total number of features of the final models are reported in Table 1. Summary plots of AUC performances obtained with L1, XGB and RF for all data groups and historical windows of 120 and 180 days are shown in Supplementary information, Figs. S1 and S2.

**Table 1:** Best AUC values across the cohorts and outcome metrics obtained with different data groups with L1, RF and XGB models for a historical window length of 180 days.

Outcome	Cohort	All+DRG	All	Meds	Patho	Basic	Expert$^*$	Pruned Final Model
		AUC	AUC	AUC	AUC	AUC	AUC	AUC (95% CIs), $N_f$
**L1**								
RA30	Children’s hospital	*0.845*	0.842	0.836	0.842	0.835	0.811	–
	Principal referral	0.746	*0.747*	0.742	*0.747*	0.741	0.726	–
	Public acute	*0.726*	*0.726*	0.719	*0.726*	0.718	0.706	–
RP 30	Children’s hospital	*0.717*	*0.717*	0.711	0.715	0.703	0.693	–
	Principal referral	*0.711*	0.709	0.707	0.709	0.705	0.685	–
	Public acute	*0.707*	0.706	0.704	0.706	0.703	0.684	–
RP30E	Children’s hospital	*0.641*	*0.641*	*0.641*	*0.641*	0.640	0.626	-
	Principal referral	*0.692*	*0.692*	0.691	*0.692*	0.691	0.687	–
	Public acute	*0.673*	*0.673*	*0.673*	*0.673*	0.672	0.671	–
**XGB**								
RA30	Children’s hospital	*0.855*	0.850	0.845	**0.851**	0.843	0.823	**0.8511 (0.8511, 0,8515), 23**
	Principal referral	*0.771*	0.766	0.761	**0.766**	0.759	0.733	**0.7662 (0.7662, 0.7663), 68**
	Public acute	*0.742*	0.739	0.731	**0.738**	0.729	0.710	**0.7382 (0.7381, 0.7382), 56**
RP 30	Children’s hospital	*0.721*	0.718	0.712	**0.720**	0.705	0.700	**0.7201 (0.7199, 0.7203), 21**
	Principal referral	*0.718*	0.714	0.711	**0.714**	0.709	0.692	**0.7125 (0.7125, 0.7126), 67**
	Public acute	*0.711*	0.710	0.709	**0.709**	0.707	0.691	**0.7086 (0.7085, 0.7086), 60**
RP30E	Children’s hospital	0.643	*0.645*	0.642	**0.641**	0.644	0.638	**0.6411 (0.6409, 0.6413), 9**
	Principal referral	*0.705*	0.698	0.697	**0.698**	0.697	0.694	**0.6974 (0.6974, 0.6975), 22**
	Public acute	*0.687*	0.678	0.677	**0.678**	0.677	0.675	**0.6784 (0.6783, 0.6784), 28**
**RF**								
RA30	Children’s hospital	*0.848*	*0.848*	0.843	0.849	0.834	0.805	–
	Principal referral	*0.760*	0.754	0.745	0.753	0.745	0.702	–
	Public acute	*0.727*	0.726	0.720	0.723	0.720	0.672	–
RP 30	Children’s hospital	0.720	*0.721*	0.713	0.720	0.709	0.661	–
	Principal referral	*0.710*	*0.710*	0.683	*0.710*	0.706	0.643	–
	Public acute	*0.711*	0.707	0.707	0.707	0.706	0.640	-
RP30E	Children’s hospital	*0.644*	0.642	0.643	0.641	0.643	0.597	–
	Principal referral	*0.694*	0.692	0.673	0.693	*0.694*	0.657	–
	Public acute	*0.675*	0.672	0.693	0.674	0.674	0.635	–

The last column reports average AUCs of pruned final models, the corresponding confidence intervals and the number of features used in the model ($N_f$). The best obtained results are shown in italic. The results obtained with the models using the selected data group and the final models are shown in bold.

#### AUC, calibration, and precision-recall plots of the final models

**Figure 2:**
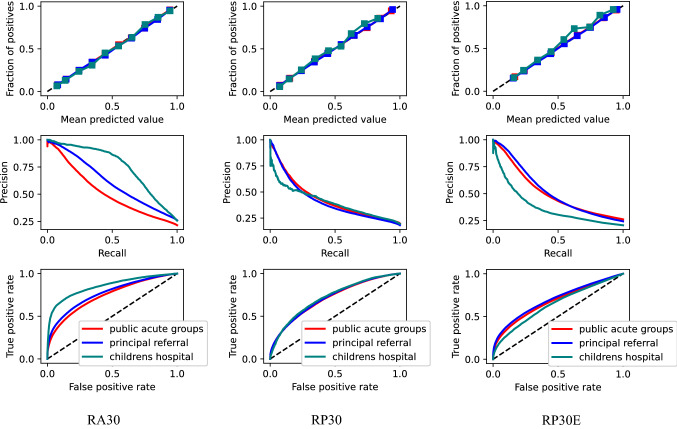
Calibration (top), PRC (middle) and AUC (bottom) plots obtained for the final models across the cohorts for the three output metrics RA30, RP30, RP30E.

Calibration plots indicate excellent calibration of the final models for all cases except for the Children’s hospital with outcomes RP30 and RP30E (Fig. 2). One of the possible reasons could be the relatively small number of samples of RP30 and RP30E for the Children’s hospital. PRC plots of the models obtained for RP30 and RP30E are characterised with exponential decreases in precision with increases in recall. Better performance was obtained for the RA30 outcome metric, in particular for the Children’s hospitals cohort. ROC plots indicate the best performance of the models was for RA30, and the poorest for RP30E. Remarkably better ROC plots are obtained for readmission models developed for the Children’s hospital.

### Model and prediction explainability

**Figure 3:**
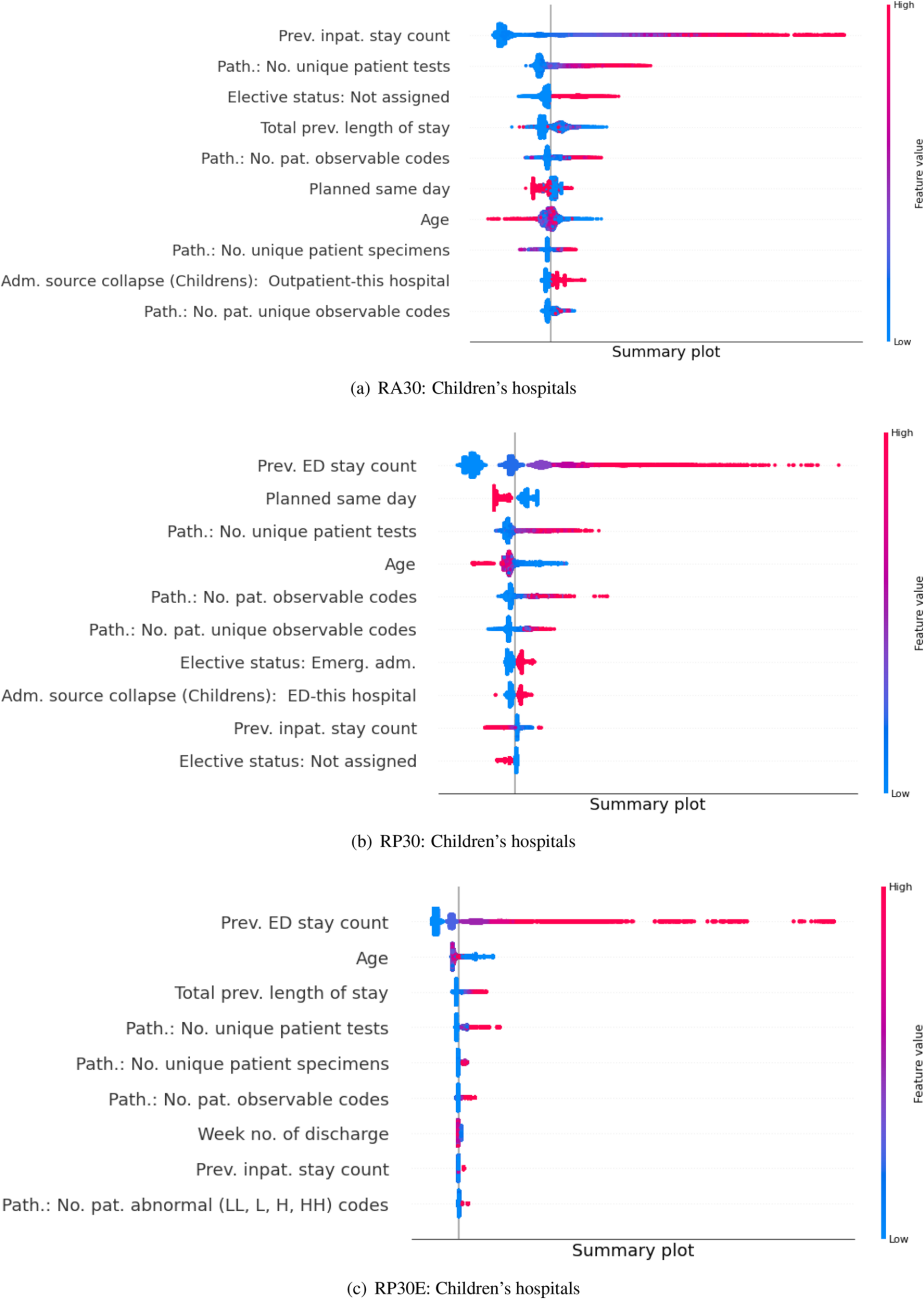
Children’s hospital: Summary plot of Shapley values computed for each patient individually in the test partition. Features are sorted top-down based on their global contribution. The distance of a dot representing a sample from the vertical line indicates its contribution. The color of a dot indicates feature value for that sample. Blue and pink color represent extreme values of the feature. Shapley values on the right side of vertical axes ‘push’ predictions towards the class 1 and those on the left side towards the class 0.

We analysed the feature contribution to the prediction for each model individually by computing the Shapley values^24^ for the whole training data (Fig. 3 and Supplement Figs. S3, S4). The length of the bar indicates the contribution of a feature: the longer the bar, the greater the contribution the corresponding feature provides to a prediction and as such will be here defined as more important. For every cohort and every outcome metric, it was observed that at least half of the features had no bar beside them, implying that they provide no contribution to predicting the output. It follows that for every cohort and every outcome metric at least half of the features make negligible contribution and as such are redundant.

Consequently, the obtained Shapley values were used as inputs into the second stage of modelling which aimed at removing features making negligible contributions from every model individually. The sizes of the pruned models, along with the corresponding AUC values, are reported in Table 1. The resultant models have at most half of the features of models with equal AUC performance presented in Table 1, showing how little is added to model discrimination by the eliminated features. The ten most important features, in descending order top to bottom, for all output metrics and cohorts and every dot representing one patient from the test sample are shown in Fig. 3 and Supplement Figs. S3, S4. For interested readers, details on the features in the final models are provided in Supplementary information (Supplementary Tables S20, S21, S22).

## Discussion

Preventing unplanned hospitalisations, including readmissions and re-presentations to the ED, is an important strategy for addressing the growing demand for hospital care. This study aimed to investigate and develop a set of deployable models for hospitals across Queensland to help identify patients with at least one chronic disease primary diagnosis at high risk of re-hospitalisation over the next 30 days. Key features of our models are that they have good discrimination, calibration and explainability.

Analysis of the AUC results leads to several remarks: (i) AUC values obtained for historical data windows of 180 days are systematically higher than for 120 days across all presented scenarios, which implies that a longer data history contributes to improved predictive performance; (ii) Adding more features does not always substantially increase predictive performance; (iii) The maximum AUC value can be systematically obtained with models that do not require all available data; (iv) The biggest fluctuation of AUC for different model sizes occurs for RP30 for all cohorts, and especially for the Children’s hospital; (v) The simple ‘Expert’ model has systematically the worst AUC performance, especially for RP30.

Based on the reported results (Table 1), we observed that predicting readmissions to hospital (RA30) was slightly easier than predicting re-presentations to ED after an inpatient stay (RP30), which was slightly easier again than predicting re-presentations to ED after an ED stay (RP30E). It was also seen that readmissions (RA30) and re-presentations to ED after an inpatient stay (RP30) were easiest to predict at the Children’s Hospital, whereas re-presentations to ED after an ED visit (RP30E) were easiest to predict at the larger Principal Referral hospitals.

A notable observation was the miscellaneous difference of less than 0.01 in AUC between XGB models obtained for ‘All-inclusive DRG model’ and ‘Patho’ data groups, while the difference in model size was almost double. Considering that big models are prone to overfitting and as such can be unstable, we selected models obtained for the ‘Patho’ data group for further analysis. Also, a difference of less than 0.01 is quite likely not statistically significant, which additionally supports the selection of models obtained for the ‘Patho’ data group. Importantly, the results obtained for the data group ‘Basic’ are comparable to the best AUC results. This implies that there is a subset of features contained in ED and inpatient datasets alone which provides useful information for predicting hospitalisation risk. Additional features obtained from pathology and medication in-hospital datasets made only modest performance improvements.

Regarding the influence of the number of features on performance, differences were seen in both AUC and calibration. For example, with the simple “Expert” models, AUC was consistently lowest, roughly 0.02 lower than “Basic” using logistic regression and XGB for inpatient outcomes (RA30, RP30; and more using Random Forest) but less for the ED outcome (RP30E). With logistic regression, the calibration of ‘Expert’ models was quite poor, but acceptable for some (but not all) of the XGB models. The XGB approach for RP30E using the “Expert” predictors might be seen as a possible simpler alternative, albeit with slightly lower AUC performance, for the Principal referral and Public acute cohorts. But, using models obtained for the same data group (i.e., Patho) will make use of the algorithm easier for clinicians who work often in multiple hospitals in the state.

The discrimination performance of readmission risk models has been summarised extensively^1,6,7,11–14^. However, it has been recognised that comparing the predictive performance of models across different studies may be challenging due to differences in the target population^11^. Each target population has its own inherent specificities, so when comparing models targeting different populations, a comparison may not be very informative. Though not directly comparable, we discuss current state-of-the-art results in this domain.

In the systematic reviews undertaken by Artetxe et al.^11^ and Zhou et al.^14^ they report AUC values ranging from 0.54 to 0.92 and from 0.55 to 0.80, respectively. Artetxe et al.^11^ state that the discrimination of the models is not comparable since they are greatly influenced by the population object of the study as well as factors such as readmission length threshold. Both findings are aligned to results of this study.

A review focusing on predictive models of readmission^13^ that specifically used ML techniques found that the performance of methods varied only slightly - similar to this study. In their review, boosted tree algorithms generally performed slightly better based on the AUC - again in line with this study and other literature showing strong performance of boosted tree algorithms for readmission risk prediction. The boosted tree algorithm is an ensemble method for regression and classification problems by combining the strengths of regression trees and boosting, and it builds the prediction model in an adaptive and iterative fashion. Besides AUC, most of the reviewed models did not report other measures, such as precision or recall, nor discussed methods to address imbalanced data. This finding highlighted the need for the current study to include complete reporting on a comprehensive list of metrics for model evaluation to enable an insightful comparison of model performance by ML methods.

**Figure 4:**
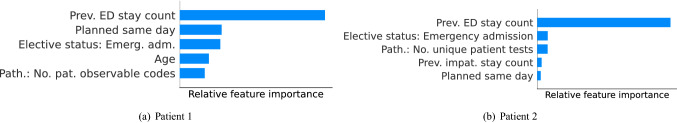
Two examples of visualizations explaining the relative importance of predictors. Each visualisation lists features in descending order based on how much they contributed to a prediction.

In light of the view that ML models often lack explainability and function as “black boxes”^25^, we produced visualisations of Shapley values which explain the relative importance of predictors contributing to each individual patient’s predicted risk score, to make it easier for end user clinicians to understand and explain model output (Fig. 4).

In the present analysis, the number of ED presentations in the previous 180 days was systematically ranked as the most important feature across all cohorts in predicting re-presentation after an inpatient stay (RP30) and an ED stay (RP30E) (Fig. 3 and Supplementary information Figs. S3, S4). Similarly, the number of inpatient stays within 180 days of discharge was the most important feature for predicting patient readmission (RA30). For both features, high values contributed towards predicting the positive class, while low values contributed towards predicting the negative class. This also explains why a model obtained using the ‘Basic’ (i.e. ED and inpatient data alone) data group had comparable performance to models comprising all types of features. High values of ‘No. pat. observable codes’ and ‘No. unique patient tests’ which counts the number of pathology results contributed remarkably in all three cohorts in predicting the positive class, which can be justified by the fact that the sicker the patient is, the more tests are performed. Age was also a relevant feature, although there was no clear distinction which values contribute to predicting class 0 and which to class 1. ‘Planned same day’ is an inpatient information field indicating whether a patient is planned to be discharge before midnight on the same day as they are admitted, and it is ranked among the most relevant predictors for RA30 and RP30 outcome metrics. Its high value push prediction towards 0 and low value towards class 1. Lastly, SEIFA index which indicates extremely disadvantaged socio-economic status is a relevant feature in predicting positive class in the RP30E outcome metric.

Similar to Yu et al.^18^ in addition to reporting AUC, we also reported precision and recall of the final models in the form of PRC plots. They show recall on the x-axis and precision on the y-axis which may better reflect the real clinical value of a predictive model since most institutions would only have resources to focus on the patients with the highest readmission risks. Considering the final models, three of the poorest PRC curves are obtained for the RP30 outcome, for which the class imbalance is much higher than the other two outcomes.

To the best of our knowledge, our study involves the largest ever sample size for developing risk models, involving electronic health records from 3 million unique patients (Supplementary Table S3). Other notable large-scale studies include modelling of 2.7 million patients in the context of 12-month readmissions^26^, and modelling of 1.3 million unique patients for an outcome of readmission in 30 days^5^. Also many other studies examine risk prediction in the context of a single health system (e.g. Amarasingham et al.^27^) and having a large sample size comprising a range of public hospital sizes reduced the risk that our sample was possibly biased.

A previous systematic review recommended that future studies should assess the relative contributions of different types of patient data to readmission risk prediction by comparing the performance of models with and without these variables in a given population^7^. Consequently, another strength of this study is that we reviewed the relative contributions of several underlying data collections including ED and inpatient hospital episodes, patient medication information and pathology test results. Unlike the findings of Zhou et al.^14^ who report that ‘laboratory tests’ and ‘medication’ variables had more weight in the models for cardiovascular disease and medical condition-related readmissions, we found only trivial performance differences between them. However, a limitation of our study was that due to the scale, the data collections we considered were all based on EHR. Broader datasets covering other aspects of a patient’s lifestyle (e.g. psychosocial factors) would enable the relative contribution of these to be assessed. Variables on functional mobility and independence may improve overall performance but are unlikely to be embedded widescale across all patients as part of standardised data collection. For example, in a study from a single hospital in the context of paediatric readmissions, the authors report that written discharge documentation and clinical information improved unplanned rehospitalisation prediction accuracy compared with administrative data alone^3^. Specifically, they found that English language proficiency, the completeness of discharge documentation, and timeliness of issuing the discharge summary added value to prediction models. Similar to the findings of this study, their study, which assessed three underlying datasets, found the highest prediction accuracy was obtained using a XGB model (C-statistic=0.654), followed closely by RF^3^. Both were superior to a LR approach. A study similar in scope to the one reported in this paper has been reported^9^. An overall model c-statistic of 0.72 was achieved. The developed model was successfully integrated into the statewide Maine Health Information Exchange to identify patient readmission risk upon admission and daily during hospitalization or for 30 days subsequently, providing daily risk score updates. Regarding the difference in predictive performance using an ML approach over LR, we found a minor benefit to using the ML approach when the same predictors were available to each approach. It is unclear whether additional work with the logistic regression models (e.g., cubic splines instead of powers for continuous predictors) would close the gap.

AUROCs and AUPRCs, while important, do not translate directly to the clinical context. Though, they are the only way to evaluate algorithms in the development and validation phases. The next step, which is not in the scope of this paper, involves using the calibration curve to translate probabilistic output values of the final models into risk groups by using quantiles to create user-defined risk thresholds e.g. at the hospital level. This should be done in consultation with clinicians. Defining any threshold and conducting the performance assessment for arbitrary threshold values is out of the scope of this study and is planned as the first step of the implementation phase. Setting risk thresholds and assessing the performance for those need not be indicative of performance for other choices of thresholds. To gain an insight how our models would perform in clinically meaningful terms we computed the total (i.e., the expected value) predicted and actual readmissions/re-presentations in our Test dataset for each cohort and outcome (Supplementary Table S27).

The models developed for this study have evolved as a statewide response to the clinical imperative to improve healthcare planning and quality of service. They fit well to the existing workflows and protocols of hospitals supported by electronic health records, however, they are subject to adjustment due to coding changes that may occur in the underlying data. The models provide real-time support in preventing unplanned patient returns to hospital, improving equality in health care access based on actual need, and facilitating individual-based health care planning through the provision of risk scores to treating practitioners. Beside the predicted outcome, the developed models also provide insight into the main factors driving the prediction. Developed models address the criteria for a ‘good model’, i.e. reasonable discrimination which is comparable to the literature, excellent calibration and hence reliability in predicted risk, they use reliable data that can be easily obtained, and they employ variables that are clinically related to and validated in the populations in which use is intended.

The models have been made available to hospitals across the state and future research should focus on the evaluation of developed algorithms in clinical settings as well as exploiting the potential benefit of including other types of data such as general practice (GP) type data (BMI, smoking, alcohol consumption, pathology tests outside the hospital environment), medications acquired from within the community and not from hospital dispensing, and other lifestyle factors (e.g. exercise). Undertaking post-implementation evaluation would ensure that they are truly “good models”, i.e. models that improve clinical practice.

## Methods

### Study design

The study protocol including all methods was reviewed and approved by the Metro South Human Research Ethics Committee (ref HREC/16/QPAH/217). All methods were performed in accordance with the guidelines and regulations relevant to the ethics approval. The ethics committee approved a waiver of consent for several factors including the nature of study, the size of the dataset, the fact that the data had been previously collected, and because the research posed minimum intrusion on people’s privacy. Linked datasets were obtained from the Queensland Health Statistical Service Branch for 27 public hospitals in Queensland, Australia across the study period (1/1/2015–31/1/2020). Patients of interest in this study were all patients with at least one chronic disease primary diagnosis (refer Supplementary Table S1) recorded from a visit to a Queensland public hospital (Supplementary Table S2) within the study period. Data used for this study comprised all inpatient and emergency patient episodes, mortality data, and available in-hospital medication and pathology data for the identified patient cohort (refer Supplementary Table S3).

### Outcome variables

Three outcomes were chosen: readmission risk within 30 days following an inpatient episode (RA30), ED presentation risk within 30 days following an inpatient episode (RP30), and ED re-presentation risk within 30 days following an ED presentation (RP30E). These outcomes were chosen based on conversations with the state health department and our previous work in the field^28,29^. Binary 0/1 indicator variables were defined for each outcome of interest (RA30, RP30, RP30E) within their respective cohort. For RA30, the outcome variable for an index admission was 1 if the patient’s next admission was identified within the data and it occurred within 30 days of the end of the index stay, and 0 otherwise. Note that because of cohort exclusions, the next admission could not be a “routine” admission. For RP30, the outcome variable was 1 if there was a first ED presentation identified following an inpatient discharge and it occurred within 30 days, and 0 otherwise. For RP30E, the outcome variable was 1 if there was a subsequent ED presentation within the data and it occurred within 30 days of the separation date of the index ED stay, and 0 otherwise. Each of the outcomes is considered separately in modelling and validation procedures.

### Data description

**Figure 5:**
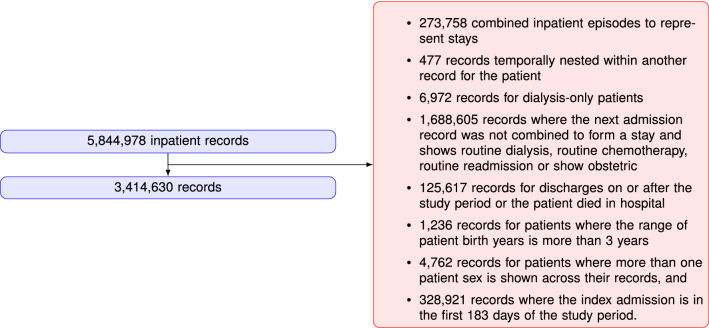
RA30 cohort selection procedure.

**Figure 6:**
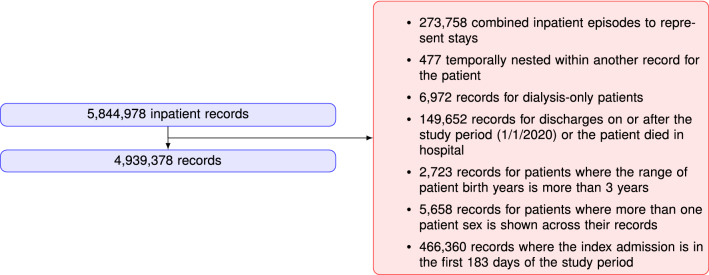
RP30 cohort selection procedure.

**Figure 7:**
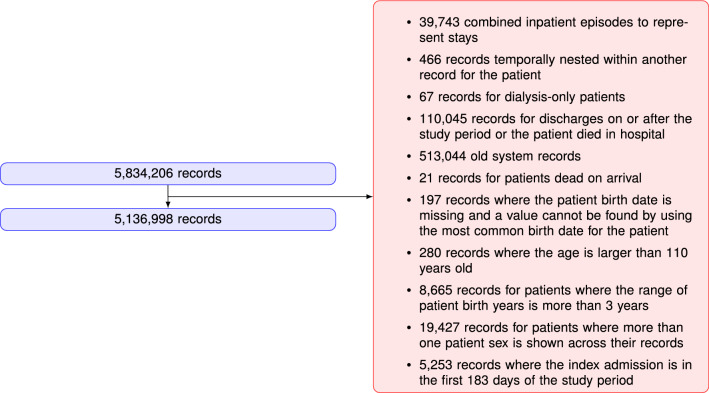
RP30E cohort selection procedure.

After filtering records with ‘routine’ stays (dialysis, chemotherapy, rehabilitation), discharges or deaths on/after 1/1/2020, admission in the first 183 days of study, more than one sex across the records, and unknown date of birth, the resulting RA30 and RP30 cohorts had 3,414,630 and 4,939,378 records respectively. For ED data, the statewide data collection changed from an old to new system early in the study period, and the recorded data are not exactly the same between the two datasets. It was decided to build the ED predictions on new system data only, although old system records were still used for capturing previous ED activity. After filtering and excluding old system records, the RP30E cohort comprised 5,136,998 records. Details of the inclusion/exclusion criteria for selecting the cohort of interest for this study are shown in Figs.  5, 6, 7.

### Hospital peer groups

The 27 state hospitals in Queensland, Australia, included in the study were clustered into three hospital groups to account for differences in services offered and patient casemix: (i) principal referral hospitals, (ii) public acute groups A, B, C, and D, and (iii) the Queensland Children’s Hospital^30^. This was used to divide the episodes into three corresponding cohorts for each outcome (refer Supplementary Table S2). Individual models were developed for each hospital group across each outcome of interest to account for differences between them.

### Predictors

Predictors used in this study were divided into the following groups: (i) *demographics*, (ii) *patient stay history*, (iii) *medication-related variables*, (iv) *pathology-related variables*, and (v) *diagnostic variables*. Demographics group predictors included patient age and Socio-Economic Indexes for Areas (SEIFA) Index of Relative Socio-economic Advantage and Disadvantage (IRSAD)^31^, anticipated to be essential predictors of risk. Square, cubic and the square root of age were created to capture a possible nonlinear relation of risk on age, particularly useful for methodologies like logistic regression which assume linear relationships. Patient history included previous inpatient counts and previous emergency visit counts (i.e. stays and presentations and total inpatient length of stay in previous interval), expected to be highly important features in predicting the patients risk. To capture a possible nonlinear relationship of the patient’s history on readmission risk, square and the square root versions of these features were created. Medication-related variables included counts of medication records such as the number of records, the number of GenericNameCodes, the number of unique GenericNameCodes, etc. Generic medication names were also mapped to Anatomical Therapeutic Chemical (ATC) level 1 categories. The ATC classification is a multilevel hierarchy, and can be used to match generic medication names to 14 categories at level 1 and 90 categories at level 2. Pathology-related variables included five predictors defined based on counts of the “Abnormal Flag” field as abnormal (“HH”, “H”, “L”, “LL”) or not normal (“notN”). Additional pathology predictors were defined based on mapping observable free text to departments (e.g., Chemical Pathology, Cytology, Serology) using a mapping table provided by Pathology Queensland. Predictors were defined as the count of observable records matched to each department where AbnormalFlag was not normal. Binary 0/1 indicators were also defined for each department where 1 indicated the corresponding counting predictor for that department was at least one. All predictors related to patient history, medication, and pathology-related variables were created for historical windows of 120 days and 180 days.

Interested readers are referred to Supplementary information for details related to the received data, data grouping and feature engineering across all outcome metrics (Supplementary Tables S1–S22).

### Prediction models

Following initial exploratory modelling, three modelling approaches (LR, RF, and XGB) were chosen for model development and validation. Logistic regression with *l*1 regularization factor (L1), often referred to as LASSO, was chosen because of its established efficacy in solving such prediction problems. An advantage of LR is that predictions can be calculated using straightforward mathematics, which makes implementation of models in a production environment easier than some other approaches. Considering that large predictor sets were considered, where many predictors may not be highly useful for predicting an outcome metric, LASSO regression was applied to perform both variable selection and regularisation in order to increase model parsimony and to enhance the predictive accuracy and interpretability of the resulting statistical models. Two tree-based approaches, RF and XGB, were employed because of their established superiority in pattern recognition from large complex data. Besides being powerful, they also allow insight into the model structure, unlike some other state-of-the-art ML approaches such as deep neural networks. Models were trained on data from January 2016 to December 2018. Hyperparameters of each of the models were tuned with stratified 3-fold cross validation using the training partition with an objective function to maximise AUC. Hyperparameters were optimised with grid search incorporated with 3-fold cross validation on training data. Once found, the best sets of parameters were used to refit the models on the complete training data. Details of hyperparameter grids and the best parameters are provided in Supplementary information (Supplementary Tables S24–S26).

### Explainability of tree-based models

In contrast to regression models which enable direct insight into the model, AI/ML models require an additional element—a so called “explainer”—to explain the association between the input and predicted output. In this study we used an explainer based on Shapley values introduced in Lundberg et al.^24^ A Shapley value $s_{i,j}$ for the sample *i* and predictor $x_{j}$ can be interpreted as the contribution of the predictor $x_{j}$ in predicting class 0 or 1 of the sample *i*, compared to the baseline prediction from a predictorless model computed as the proportion of samples belonging to a class of interest. As such, it can take positive and negative values. Positive can be interpreted as driving the prediction toward class 1 and negative toward class 0. We created the explainer for the selected model to analyse global predictor importance in predicting the class 1 (i.e., patients at high risk of readmission). Besides global importance, the explainer was used to explain predictions of unseen individual instances to provide insight into which predictors contributed the most in predicting the obtained output.

### Modelling scenarios

To investigate model performance and relevance of different data in predicting RP30, RA30 and RP30E, we conducted exhaustive modelling with L1, XGB and RF classifiers for all combinations of data type, historical window, and cohorts. Specifically, we investigated models which used predictors from each of the following data groups:

(i) administrative data from the inpatient and ED datasets, denoted in the results as the ‘Basic’ model;
(ii) administrative data and medications, denoted in the results as the ‘Meds’ model;
(iii) administrative data and pathology, denoted in the results as the ‘Patho’ model;
(iv) administrative data, medications, and pathology, denoted in the results as the ‘All-inclusive’ model;
(v) administrative data, medications, pathology, ICD top 20 diagnosis, DRG and Charlson Index, denoted in the results as the ‘All-inclusive DRG’; and
(vi) a simple regression model comprising predictors suggested by senior health care administrators, denoted as the ‘Expert’ model (comprised of the following predictors: previous inpatient visit counts, previous emergency visit counts (stays and presentations), age, SEIFA Index of IRSAD^31^ and indicator for stay longer than a day).

To account for differences in services offered and patient casemix, separate models were developed for each of the three hospital peer-groups. Two different historical window lengths of 120 and 180 days were investigated for all cohorts, which resulted in 360 possible combinations. Including the number of combinations possible based on specified hyperparameters grid, more than 2500 models were explored per outcome metric.

### Retrospective evaluation

Following the TRIPOD recommendations for validating predictive models^23^, models were validated using temporal validation approach such that stays beginning in the last year of the study period (i.e. stays beginning 1/1/2019 or later) were used as a test data, while the reminder of the data was used for model training. This approach was used for all cohorts and all outcome metrics. To evaluate the predictive performance of the models on test data we used AUC as the main model evaluation metric and calibration curves to choose the final models. PRC curves obtained for the final models are provided as complementary information to the ROC curves.

#### Statistical analysis

To measure variability of the estimates in the final models, means and 95% Confidence Intervals (CIs) reported are obtained from 1000 bootstrapped samples drawn with replacement from the test set. CIs are computed with two paired t-test.

### Software

All models were trained in Python 3.6. We employed the sklearn package to implement RF and L1 and the XGBoost package to implement the XGB classifier. For hyperparameter tuning we used the GridSearchCV function from Scikit-learn’s model_selection package. Shapley values used for explanations of the model were calculated with the SHAP package (https://github.com/slundberg/shap). The analysis, training and tests were performed with custom code written in Python 3.6. For visualisation of some results, the R package ggplot2 was used. Statistical analysis of model performance was conducted with scipy.stats Python module.

## Supplementary Information

Supplementary Information.

## Data Availability

The datasets analysed in the current study are not publicly available. Access to the original data can be provisioned by submitting a new application for appropriate regulatory approvals. Correspondence and requests should be addressed to A.B. (email: aida.brankovic@csiro.au).

## Code availability

All procedures and implementations are described in details within the “Methods” section and supplement to allow identical replication. Any inquiry regarding the technical details of the specific models can be made by relevant parties to the corresponding author. The code used for model development and validation is available from the corresponding author on reasonable request for non-commercial purposes.
