# Supplementary material for: Identifying patients at risk of unplanned re-hospitalisation using statewide electronic health records
Source: Sci Rep. 2022 Oct 5;12:16592. doi: 10.1038/s41598-022-20907-z (PMC9534931; doi:10.1038/s41598-022-20907-z)
Supplement: Supplementary file 1 — Supplementary Information. [file 41598_2022_20907_MOESM1_ESM.pdf]

Supplementary information for

Identifying Patients at Risk of Unplanned Re-Hospitalisation using Statewide Electronic Health Records

Aida Brankovic[1,*], David Rolls[2,+], Justin Boyle[1,+], Philippa Niven[2], and Sankalp Khanna[1]

[1]CSIRO, The Australian e-Health Research Centre, Brisbane, 4029, Australia

[2]CSIRO, The Australian e-Health Research Centre, Melbourne, 3052, Australia

[*]Corresponding author aida.brankovic@csiro.au

[+]These authors had the same contribution

Appendix A **Received data and hospital grouping** **3**

Appendix B **Predictors used in the study** **5**

Prev. inpat. stay count (for RA30, RP30) 12
Prev. inpat. stay count (for RP30E) 12
Prev. ED stay count 13
Related variable: ED_presentations_qhapdc_stays_prev_1_180d_counts 13
Prev. ED count 13
Related variable: ED_presentations_edcedis_stays_prev_1_180d_counts 13
Prev. inpat. stay count 13
Related variable: qhapdc_LOS_prev_1_180d 13
Sex 13
Marital status 14
Age 14
Indigenous status 14
Senior flag 14
IRSAD decile 14
Total prev. length of stay 14
Related variable: LOS 14
Sin/Cos of week number of discharge 14
Adm source collapse 14
Adm source collapse 16
Adm unit 16
Care type 18
Routine dialysis 18
DRG_70_category_{ZZZ} 18

Appendix D **Demographic summary by dataset and readmission/representation status** **19**

Appendix E **Predictors included in the pruned final models** **22**

Appendix F **Hyperparameter tuning** **25**

Appendix G **AUC summary results** **27**

Appendix H **Explainability plots** **30**

Appendix I **Calibration plots for Expert models** **32**

Appendix J **Expected number of outcomes and fraction of positives across the outcome metrics and cohorts** .. **34**

List of Tables

Supplementary Table S1 List of ICD-10 primary diagnosis codes used to identify chronic disease patients for the study period 3
Supplementary Table S2 Grouping of hospitals by peer group 3
Supplementary Table S3 Summary of data received 4
Supplementary Table S4 Admission Source Mapping Table (Principal Referral and Public Acute Hospitals) 5
Supplementary Table S5 Admission Source Mapping Table (Children's Hospital) 5
Supplementary Table S6 Admission Unit Mapping Table 5
Supplementary Table S7 Care type Mapping Table 7
Supplementary Table S8 Standard Ward Code Mapping Table 7
Supplementary Table S9 List of available and derived predictors used for model development for RA30 and RP30 8
Supplementary Table S10 List of available and derived predictors used for model development for RP30E 9
Supplementary Table S11 Abbreviations 10
Supplementary Table S12 Counts of candidate predictors across the hospital peer groups and outcome metrics 11
Supplementary Table S13 Admission Source Mapping Table (Principal Referral and Public Acute Hospitals) 15
Supplementary Table S14 Admission Source Mapping Table (Children's Hospital) 16
Supplementary Table S15 Admission Unit Mapping Table 16
Supplementary Table S16 Care type Mapping Table 18
Supplementary Table S17 Demographic summary for RA30, by modelling dataset and representation status 19
Supplementary Table S18 Demographic summary for RP30, by modelling dataset and representation status 20
Supplementary Table S19 Demographic summary for RP30E, by modelling dataset and representation status 21
Supplementary Table S20 Children's hospitals: Features used in the final models across the outcome metrics 22
Supplementary Table S21 Principal referral hospitals: Features used in the final models across the outcome metrics 23
Supplementary Table S22 Public acute hospitals: Features used in the final models across the outcome metrics 24
Supplementary Table S23 Hyperparameters grid 25
Supplementary Table S24 Final sets of hyperparameters across the hospital peer groups and RA30 outcome metric for data group Patho and historical window 180 days 25
Supplementary Table S25 Final sets of hyperparameters across the hospital peer groups and RP30 outcome metric for data group Patho and historical window 180 days 26
Supplementary Table S26 Final sets of hyperparameters across the hospital peer groups and RP30E outcome metric for data group Patho and historical window 180 days 26
Supplementary Table S27 Expected number of outcomes and fraction of positives across the outcome metrics and cohorts computed on test data. 34

List of Figures

Supplementary Figure S1 L1 model: AUC as a function of model size across the cohorts and outcome metrics for different data groups 28
Supplementary Figure S2 RF and XGB models: AUC performance for different data groups across the cohorts and outcome metrics 29
Supplementary Figure S3 Principal referral hospitals: Summary plot of Shapley values computed for each patient individually in the test partition. Features are sorted top-down based on their global contribution. The distance of a dot representing a sample from the vertical line indicates its contribution. The colour of a dot indicates feature value for that sample. Blue and pink colour represent extreme values of the feature. Shapley values on the right side of vertical axes 'push' predictions towards the class 1 and those on the left side towards the class 0. 30
Supplementary Figure S4 Calibration plots obtained for Expert models and logistic regression across the cohorts and prediction outcomes. 32
Supplementary Figure S5 Calibration plots obtained for Expert models and XGB across the cohorts and prediction outcomes. 32
Supplementary Figure S6 Calibration plots obtained for Expert models and RF across the cohorts and prediction outcomes. 33

Appendix A Received data and hospital grouping

Patients of interest in this study were all patients with at least one chronic disease primary diagnosis (refer to the list of chronic disease ICD 10 codes below) recorded from a visit (ED presentation or inpatient admission) to a Queensland public hospital in the timeframe 1/1/2015 - 31/1/2020. Table S1 lists ICD-10 primary diagnosis codes used to identify chronic disease patients for the study period.

Supplementary Table S1 List of ICD-10 primary diagnosis codes used to identify chronic disease patients for the study period

DESCRIPTION	ICD CODE
Neoplasms	C00–D48
Diseases of the blood and blood–forming organs and certain disorders involving the immune mechanism	D50–D89
Endocrine, nutritional and metabolic diseases	E00–E90
Mental and behavioural disorders	F00–F99
Diseases of the nervous system	G00–G99
Diseases of the eye, adnexa, ear and mastoid process	H00–H95
Diseases of the circulatory system	I00–I99
Diseases of the respiratory system	J00–J99
Diseases of the digestive system	K00–K93
Diseases of the skin and subcutaneous tissue	L00–L99
Diseases of the musculoskeletal system and connective tissue	M00–M99
Diseases of the genitourinary system	N00–N99
Care Involving Dialysis	Z49

The scope of the study covered the development and validation of the models across 27 public hospitals of different sizes. Separate models were developed for 3 hospital peer groups to account for differences in services offered and patient casemix – i) principal referral hospitals; ii) public acute groups A, B, C, and D; and iii) the Queensland Children's Hospital. The peer grouping for each hospital is presented in Table S2 below.

Supplementary Table S2 Grouping of hospitals by peer group

HOSPITAL	CURRENT PEER GROUP
Queensland Children's Hospital	Specialist children's hospital
Gold Coast University Hospital	Principal referral hospitals
Sunshine Coast University Hospital	Principal referral hospitals
The Townsville Hospital	Principal referral hospitals
Princess Alexandra Hospital	Principal referral hospitals
The Prince Charles Hospital	Principal referral hospitals
Royal Brisbane & Women's Hospital	Principal referral hospitals
Logan Hospital	Public acute group A hospitals
Cairns Base Hospital	Public acute group A hospitals
Ipswich Hospital	Public acute group A hospitals
Bundaberg Hospital	Public acute group A hospitals
Toowoomba Hospital	Public acute group A hospitals
Redcliffe Hospital	Public acute group A hospitals
Nambour Hospital	Public acute group A hospitals
Rockhampton Base Hospital	Public acute group A hospitals
Mackay Base Hospital	Public acute group A hospitals
Hervey Bay Hospital	Public acute group A hospitals
Queen Elizabeth II Jubilee Hospital	Public acute group A hospitals
Caboolture Hospital	Public acute group B hospitals
Mount Isa Hospital	Public acute group B hospitals
Redland Hospital	Public acute group B hospitals
Gladstone Hospital	Public acute group B hospitals
Gympie Hospital	Public acute group B hospitals
Maryborough Hospital	Public acute group B hospitals
Robina Hospital	Public acute group B hospitals
Innisfail Hospital	Public acute group C hospitals
Beaudesert Hospital	Public acute group D hospitals

Inpatient, Emergency, in-hospital Medications, Pathology and Mortality data were received for 27 hospitals across the study period (1/1/2015 – 31/1/2020) as presented in Table S3 below.

Supplementary Table S3 Summary of data received

ORIGINAL DATA	OBSERVATIONS/ROWS	VARIABLES/COLUMNS	UNIQUE PATIENTS
Inpatient (QHAPDC)	5,844,978	22	1,401,005
Inpatient (Standard unit codes)	6,338,809	3	1,401,005
Inpatient (Morbidity codes)	29,999,918	6	1,401,005
Emergency (EDC)	5,317,062	16	1,557,913
Emergency (EDIS)	517,144	15	332,212
Death Registry	105,917	3	105,917
Medications	18,417,967	n/a (XML data)	527,877
Pathology	475,287,387	n/a (XML data)	1,437,364

Appendix B Predictors used in the study

This section describes the implementation of predictor variables in the final models. Due to data sparsity, the original categories in some categorical variables were collapsed. These are indicated with a mapping table between the original and collapsed categories (Table S4-S8).

The list of available and derived predictors used for model development for RA30, RP30 and RP30E for all three hospital peer groups are reported in Table S9 and S10.

Supplementary Table S4 Admission Source Mapping Table (Principal Referral and Public Acute Hospitals)

ADM. SOURCE	ADM SOURCE COLLAPSED
emergency department - this hospital	emergency department - this hospital
outpatient department - this hospital	outpatient department - this hospital
routine readmission not requiring referral	routine readmission not requiring referral
Admitted patient transferred from another hospital	Admitted patient transferred from another hospital
private medical practitioner (not psychiatrist)	private medical practitioner (not psychiatrist)
born in facility	born in facility
Other	other
residential aged care service	residential aged care service
boarder	boarder
Non-Admitted patient referred from another hospital	Non-Admitted patient referred from another hospital
Community service	Other
episode change	Other
Planned emergency	Other
other health care establishment	Other
organ procurement	Other
private psychiatrist	Other

Supplementary Table S5 Admission Source Mapping Table (Children's Hospital)

ADM. SOURCE	ADM SOURCE COLLAPSED (CHILDREN'S)
emergency department - this hospital	emergency department - this hospital
outpatient department - this hospital	outpatient department - this hospital
routine readmission not requiring referral	Other
Admitted patient transferred from another hospital	Admitted patient transferred from another hospital
private medical practitioner (not psychiatrist)	private medical practitioner (not psychiatrist)
born in facility	Other
Other	Other
residential aged care service	Other
boarder	Other
Non-Admitted patient referred from another hospital	Other
Community service	Other
episode change	Other
Planned emergency	Other
other health care establishment	Other
organ procurement	Other
private psychiatrist	Other

Supplementary Table S6 Admission Unit Mapping Table

ADM. UNIT	ADM. UNIT DESCRIPTION	ADM UNIT COLLAPSED
ADDU	Alcohol & Drug Dependency	ADDU
CARD	Cardiology	CARD
CARS	Cardiac Surgical	CARS_CART
CART	Cardio Thoracic Surgical	CARS_CART
CCU	Coronary Care Unit	CCU
EMED	Emergency Medicine	EMED
ENDO	Endocrinology and Diabetes	ENDO
ENT	Ear Nose and Throat	ENT_MAXI
MAXI	Maxillofacial	ENT_MAXI
GAST	Gastroenterology	GAST

ADM. UNIT	ADM. UNIT DESCRIPTION	ADM UNIT COLLAPSED
GENM	General Medical	GENM_GENO
GENO	General Medical/Surgical	GENM_GENO
GENS	General Surgical	GENS
HAEM	Haematology	HAEM
ICU	Intensive Care	ICU
IMMU	Immunology	IMMU_RHEU
RHEU	Rheumatology	IMMU_RHEU
NEUR	Neurology	NEUR
NSUR	Neurosurgery	NSUR
BIRT	Birthing Unit	OB (Birth/Maternity)
OBST	Obstetric/Maternity	OB (Birth/Maternity)
GYNA	Gynaecology Medical	all other (OB/GYN)
GYNC	Gynaecology Medical/Surgical	all other (OB/GYN)
GYNS	Gynaecology Surgical	all other (OB/GYN)
OBSG	Obstetrics/Gynaecology	all other (OB/GYN)
GYNO	Gynaecology Oncology	Oncology (Med.,Surg., Rad.)
ONCM	Oncology - Medical	Oncology (Med.,Surg., Rad.)
ONCS	Oncology - Surgical	Oncology (Med.,Surg., Rad.)
ONCR	Oncology - Radiation	Oncology (Med.,Surg., Rad.)
OPHT	Ophthalmology	OPHT
ORTH	Orthopaedic	ORTH
HIGH	High Dependency Unit	Other
HIV	AIDS/HIV Unit	Other
NEOI	Neonatal Intensive Care	Other
DENT	Dental/Oral	Other
INFE	Infectious Diseases	Other
DAY	Day Surgery	Other
DERM	Dermatology	Other
ENDS	Endoscopy	Other
GERI	Geriatric	Other
HYPE	Hyperbaric	Other
RADI	Radiology	Other
SPIN	Spinal Injuries	Other
PAIN	PAIN	Other
ANAE	Anaesthetic/Pain Management	Other
OUTS	Outpatients - Specialists	Other
RESP	Respite	Other
SLEP	Sleep Centre	Other
WOCH	Women and Child Health Medical/Surgical	Other
PEEN	Paediatric - Ear Nose and Throat	PAED (ENT)
NEON	Neonatology	PAED (General)
PEGE	Paediatric - General	PAED (General)
PEDE	Paediatric - Dental/Oral	PAED (Other Internal/External)
PEDR	Paediatric - Dermatology	PAED (Other Internal/External)
PEDV	Paediatric - Developmental	PAED (Other Internal/External)
PEMA	Paediatric - Maxillofacial	PAED (Other Internal/External)
PEOP	Paediatric - Ophthalmology	PAED (Other Internal/External)
PEOR	Paediatric - Orthopaedic	PAED (Other Internal/External)
PEPL	Paediatric - Plastic/Reconstructive Surgery/Burns	PAED (Other Internal/External)
PECA	Paediatric - Cardiology	PAED (Other Internal/External)
PECS	Paediatric - Cardiac Surgical	PAED (Other Internal/External)
PECY	Paediatric - Cystic Fibrosis	PAED (Other Internal/External)
PEED	Paediatric - Endocrinology/Diabetics	PAED (Other Internal/External)
PEGA	Paediatric - Gastroenterology	PAED (Other Internal/External)
PENE	Paediatric - Nephrology	PAED (Other Internal/External)
PENL	Paediatric - Neurology	PAED (Other Internal/External)
PERE	Paediatric - Respiratory Medicine	PAED (Other Internal/External)
PERH	Paediatric - Rheumatology	PAED (Other Internal/External)
PESP	Paediatric - Spinal	PAED (Other Internal/External)
PEUR	Paediatric - Urology	PAED (Other Internal/External)
PALG	Palliative - General	all palliative
PALL	Palliative - Designated Unit	all palliative
PEON	Paediatric - Oncology	PEON
PENS	Paediatric - Neurosurgery	PICU, PESU, PENS, PELT
PEIC	Paediatric - Intensive Care	PICU, PESU, PENS, PELT
PELT	Paediatric - Liver Transplant	PICU, PESU, PENS, PELT
PESU	Paediatric - Surgery	PICU, PESU, PENS, PELT
PLAS	Plastic/Reconstructive Surgery/Burns	PLAS
PYAA	Psychiatric Adult Acute Unit	PSYCH (Adult)
PYAQ	Psychiatric Adult Ext - Acquired Brain Damage Unit	PSYCH (Adult)
PYAW	Psychiatric Adult Special Care Suite	PSYCH (Adult)
PYDD	Psychiatric Adult Extended - Dual Diagnosis Unit	PSYCH (Adult)
PYET	Psychiatric Adult Extended - Treatment Rehab Unit	PSYCH (Adult)
PYFA	Psychiatric Forensic Acute	PSYCH (Adult)
PYGE	Psychiatric Older Persons -Acute	PSYCH (Adult)
PYPG	Psychiatric Adult Extended - Psychogeriatric Unit	PSYCH (Adult)
PYRA	Psychiatric Adult Residential	PSYCH (Adult)
PYSH	Psychiatric Adult Ext - Extended High Security Unit	PSYCH (Adult)
PYSM	Psychiatric Adult Ext - Extended Secure Medium Unit	PSYCH (Adult)

ADM. UNIT	ADM. UNIT DESCRIPTION	ADM UNIT COLLAPSED
PYYW	Psychiatric Adolescent Acute Unit in Adult Ward	PSYCH (Adult)
PYOA	Psychiatric Young Persons (Youth) Acute Unit	PSYCH (Child/Adolescent)
PYYA	Psychiatric Young Persons (Youth) Acute Unit	PSYCH (Child/Adolescent)
PYCA	Psychiatric Child Acute Unit	PSYCH (Child/Adolescent)
PYCW	Psychiatric Child Acute Unit in Paediatric Ward	PSYCH (Child/Adolescent)
REHA	Rehabilitation -Geriatric	Rehab-All
REHD	Rehabilitation -Designated Unit	Rehab-All
REHG	Rehabilitation -General	Rehab-All
RENA	Renal/Nephrology	RENA
THOR	Thoracic/Respiratory Medical	Thoracic (Med., Surg.)
THOS	Thoracic Surgery	Thoracic (Med., Surg.)
TRAB	Transplantation Unit -Bone	All transplant
TRAH	Transplantation Unit -Heart/Lung	All transplant
TRAL	Transplantation Unit -Liver	All transplant
TRAM	Transplantation Unit -Bone Marrow	All transplant
TRAP	Transplantation Unit -Pancreas	All transplant
TRAR	Transplantation Unit -Renal	All transplant
UROL	Urology	UROL
VASC	Vascular	VASC
PYAM	Psychiatric Adult	PSYCH (Adult)

Supplementary Table S7 Care type Mapping Table

CARE TYPE	CARE TYPE COLLAPSE	CARE TYPE COLLAPSED
Acute	Acute	Acute
Mental Health	Mental Health	Mental Health
Newborn	Newborn	Newborn
Palliative care	Palliative	Palliative
Boarder	Other care	Other care
Rehabilitation care	Rehabilitation	Other care
Palliative - according to a designated program	Palliative	Palliative
Geriatric Evaluation and Management	Other care	Other care
Maintenance	Other care	Other care
Palliative - delivered in a designated unit	Palliative	Palliative
Psychogeriatric	Other care	Other care
Palliative - principal clinical intent	Palliative	Palliative
Other care	Other care	Other care
Rehabilitation - principal clinical intent	Rehabilitation	Other care
Organ procurement	Other care	Other care

Supplementary Table S8 Standard Ward Code Mapping Table

STND WARD CODE	STND WARD CODE COLLAPSED
CCU4	CCU_456
CCU5	CCU_456
CCU6	CCU_456
CHEM	CHEM
CIC4	CIC_456
CIC5	CIC_456
CIC6	CIC_456
DIAL	DIAL
EDSS	EDSS
EMER	EMER
HOME	HOME
ICU4	ICU_456
ICU5	ICU_456
ICU6	ICU_456
MATY	MATY
MENA	MENA
MENN	MENN
MIXC	MIX_CG
MIXG	MIX_CG
NSV4	NSV_456
NSV5	NSV_456
NSV6	NSV_456
OBSV	OBSV
PAED	PAED
SNAP	SNAP
STKU	STKU
TRNL	TRNL

Supplementary Table S9 List of available and derived predictors used for model development for RA30 and RP30

PREDICTOR NAME	DESCRIPTION	PREDICTOR NAME	DESCRIPTION
Prev. inpat. stay count (120 d)	Previous inpatient visit counts	Adm. unit: UROL	
Prev. inpat. stay count (120 d) ^2		Adm. unit: VASC	
Prev. inpat. stay count (180 d)		Care type: Acute	
Prev. inpat. stay count (180 d) ^2		Care type: Mental Health	
Prev. ED stay count (120 d)	Previous emergency visit counts (stays and presentations)	Care type: Newborn	
Prev. ED stay count (120 d) ^2		Care type: Other care	
Prev. ED stay count(120 d) ^3		Care type: Palliative	
Prev. ED count (180 d)		Elective status: Emerg. adm.	
Prev. ED count (180 d) ^2		Elective status: Not assigned	
Prev. ED count (180 d) ^3		Adm. source collapse (Childrens): Admitted transfer in	
Age	Patient demographic information	Adm. source collapse (Childrens): Boarder	
Age^2		Adm. source collapse (Childrens): ED-this hospital	
Age^3		Adm. source collapse (Childrens): Other	
Sex: Male		Adm. source collapse (Childrens): Outpatient-this hospital	
Marital status: Divorced		Adm. source collapse (Childrens): Pvt. med. practit. (not Psychr.)	
Marital status: Married/de facto		Senior (age>=65) flag	
Marital status: Not stated/unknown		Week no. of discharge	
Marital status: Separated		Sine of week no. of discharge	
Marital status: Widowed		Sine of week no. of discharge (senior only)	
Indigenous status: Indigenous		Cosine of week no. of discharge	
Indigenous status: Not indigenous/unknown		Cosine of week no. of discharge (senior only)	
SEIFA IRSAD decile: 1		Meds: No. pat. records (120 d)	Counts from Medications data
SEIFA IRSAD decile: 2		Meds: No. pat. records (180 d)	
SEIFA IRSAD decile: 3		Meds: No. ATC A pat. records (120 d)	Medication in previous 120-180 days - ATC level 2 hierarchy
SEIFA IRSAD decile: 4		Meds: No. ATC B pat. records (120 d)	
SEIFA IRSAD decile: 5		Meds: No. ATC C pat. records (120 d)	
SEIFA IRSAD decile: 6		Meds: No. ATC D pat. records (120 d)	
SEIFA IRSAD decile: 7		Meds: No. ATC G pat. records (120 d)	
SEIFA IRSAD decile: 8		Meds: No. ATC H pat. records (120 d)	
SEIFA IRSAD decile: 9		Meds: No. ATC J pat. records (120 d)	
SEIFA IRSAD decile: 10		Meds: No. ATC L pat. records (120 d)	
SEIFA IRSAD decile: Unknown		Meds: No. ATC M pat. records (120 d)	
Total prev. length of stay (120 d)	Total inpatient length of stay in previous interval	Meds: No. ATC N pat. records (120 d)	
Total prev. length of stay (120 d)^0.5		Meds: No. ATC P pat. records (120 d)	
Total prev. length of stay (120 d)^2		Meds: No. ATC R pat. records (120 d)	
Total prev. length of stay (180 d)		Meds: No. ATC S pat. records (120 d)	
Total prev. length of stay (180 d)^0.5		Meds: No. ATC V pat. records (120 d)	
Total prev. length of stay (180 d)^2		Meds: No. ATC A pat. records (180 d)	
routine dialysis flag (120-180 d)	Routine visit in previous 120 days or previous 120-180 days	Meds: No. ATC B pat. records (180 d)	
Adm. source: Admitted transfer in	Current visit information	Meds: No. ATC C pat. records (180 d)	
Adm. source: Non-admitted referred		Meds: No. ATC D pat. records (180 d)	
Adm. source: Boarder		Meds: No. ATC G pat. records (180 d)	
Adm. source: Born in facility		Meds: No. ATC H pat. records (180 d)	
Adm. source: Other		Meds: No. ATC J pat. records (180 d)	
Adm. source: Outpatient-this hospital		Meds: No. ATC L pat. records (180 d)	
Adm. source: Pvt. med. practit. (not Psychr.)		Meds: No. ATC M pat. records (180 d)	
Adm. source: Resid. aged care		Meds: No. ATC N pat. records (180 d)	
Adm. source: Routine readm. not requiring ref.		Meds: No. ATC P pat. records (180 d)	
Planned same day		Meds: No. ATC R pat. records (180 d)	
Adm. unit: ADDU		Meds: No. ATC S pat. records (180 d)	
Adm. unit: CARD		Meds: No. ATC V pat. records (180 d)	
Adm. unit: CARS, CART		Path.: No. unique patient tests	Pathology - number of results in previous 120-180 days
Adm. unit: CCU		Path.: No. unique patient specimens	
Adm. unit: EMED		Path.: No. pat. observable codes	
Adm. unit: ENDO		Path.: No. pat. unique observable codes	
Adm. unit: ENT, MAXI		Adm. source collapse (Childrens): Admitted transfer in	Current visit information
Adm. unit: GAST		Adm. source collapse (Childrens): Boarder	
Adm. unit: GENM, GENO		Adm. source collapse (Childrens): ED-this hospital	
Adm. unit: GENS		Adm. source collapse (Childrens): Other	
Adm. unit: HAEM		Adm. source collapse (Childrens): Outpatient-this hospital	

Adm. unit: ICU		Adm. source collapse (Childrens): Pvt. med. practit. (not Psychr.)	
Adm. unit: IMMU, RHEU		Path.: Pat. abnormal AllDep flag	Pathology - number of abnormal results in previous 120 days, by department
Adm. unit: NEUR		Path.: Pat. abnormal Chempath. flag	
Adm. unit: NSUR		Path.: Pat. abnormal Cytology flag	
Adm. unit: OB (Birth/Maternity)		Path.: Pat. abnormal Micro flag	
Adm. unit: all other (OB/GYN)		Path.: Pat. abnormal Haemotology flag	
Adm. unit: OPHT		Path.: Pat. abnormal Immu flag	
Adm. unit: ORTH		Path.: Pat. abnormal Sero flag	
Adm. unit: Oncology(Med.,Surg., Rad.)		Path.: Pat. abnormal Trans flag	
Adm. unit: Other		Path.: Pat. abnormal AllDep flag	
Adm. unit: PAED (ENT)		Path.: Pat. abnormal Chempath. flag	
Adm. unit: PAED (General)		Path.: Pat. abnormal Cytology flag	
Adm. unit: PAED (Other Internal/External)		Charlson index	Charlson Comorbidity Index
Adm. unit: all palliative			
Adm. unit: PEON			
Adm. unit: PICU, PESU, PENS, PELT			

* List of abbreviations is provided in Table 11

Supplementary Table S10 List of available and derived predictors used for model development for RP30E

PREDICTOR NAME	DESCRIPTION	PREDICTOR NAME	DESCRIPTION
Prev. inpat. stay count (120 d)	Previous inpatient visit counts	Path.: No. unique patient tests (120 d)	Pathology - number of results in previous 120 days
Prev. inpat. count (120 d) ^2		Path.: No. unique patient specimens (120 d)	
Prev. inpat. stay count (180 d)		Path.: No. pat. observable codes (120 d)	
Prev. inpat. stay count (180 d) ^2		Path.: No. pat. unique observable codes (120 d)	
Prev. ED stay count (120 d)	Previous emergency visit counts (stays and presentations)	Path.: No. pat. abnormal LL codes (120 d)	
Prev. ED stay count (120 d) ^2		Path.: No. pat. abnormal L codes (120 d)	
Prev. ED stay count (180 d)		Path.: No. pat. abnormal H codes (120 d)	
Prev. ED stay count (180 d) ^2		Path.: No. pat. abnormal HH codes (120 d)	
Age	Patient demographic information	Path.: No. pat. abnormal (LL, L, H, HH) codes (120 d)	
Age^2		Path.: No. pat. abnormal AllDep records (120 d)	Pathology - number of abnormal results in previous 120 days, by department
Age^3		Path.: No. pat. abnormal Chempath. records (120 d)	
Sex: Male		Path.: No. pat. abnormal Cytology records (120 d)	
Indigenous status: Indigenous		Path.: No. pat. abnormal Cytogenetics records (120 d)	
Indigenous status: Not indigenous/unknown		Path.: No. pat. abnormal Haematology records (120d)	
SEIFA IRSAD decile: 1		Path.: No. pat. abnormal Immu records (120 d)	
SEIFA IRSAD decile: 2		Path.: No. pat. abnormal ClinicSys records (120 d)	
SEIFA IRSAD decile: 3		Path.: No. pat. abnormal Sero records (120 d)	
SEIFA IRSAD decile: 4		Path.: No. pat. abnormal Trans records (120 d)	
SEIFA IRSAD decile: 5		Path.: No. pat. abnormal NoMatch records (120 d)	
SEIFA IRSAD decile: 6		Path.: No. unique patient tests (180 d)	Pathology - number of results in previous 180 days
SEIFA IRSAD decile: 7		Path.: No. unique patient specimens (180 d)	
SEIFA IRSAD decile: 8		Path.: No. pat. observable codes (180 d)	
SEIFA IRSAD decile: 9		Path.: No. pat. unique observable codes (180 d)	
SEIFA IRSAD decile: 10		Path.: No. pat. abnormal LL codes (180 d)	
SEIFA IRSAD decile: Unknown		Path.: No. pat. abnormal L codes (180 d)	
Senior (age>=65) flag		Path.: No. pat. abnormal H codes (180 d)	
Week no. of discharge	Current visit information	Path.: No. pat. abnormal HH codes (180 d)	
Sine of week no. of discharge		Path.: No. pat. abnormal (LL, L, H, HH) codes (180 d)	
Sine of week no. of discharge (senior only)		Path.: No. pat. abnormal AllDep records (180 d)	Pathology - number of abnormal results in previous 180 days, by department
Cosine of week no. of discharge		Path.: No. pat. abnormal Chempath. records (180 d)	

Cosine of week no. of discharge (senior only)		Path.: No. pat. abnormal Cytology records (180 d)	
Total prev. length of stay (120 d)	Total inpatient length of stay in previous interval	Path.: No. pat. abnormal Cytogenetics records (180 d)	
Total prev. length of stay (120 d)^0.5		Path.: No. pat. abnormal Haematology records (180d)	
Total prev. length of stay (120 d)^2		Path.: No. pat. abnormal Immu records (180 d)	
Total prev. length of stay (180 d)		Path.: No. pat. abnormal Micro records (180 d)	
Total prev. length of stay (180 d)^0.5		Path.: No. pat. abnormal Molecular records (180 d)	
Total prev. length of stay (180 d)^2		Path.: No. pat. abnormal ClinicSys records (180 d)	
Meds: No. pat. records	Counts from Medications data – 120 and 180 days	Path.: No. pat. abnormal Sero records (180 d)	
Meds: No. Admission pat. Records		Path.: No. pat. abnormal Trans records (180 d)	
Meds: No. Discharge pat. Records		Path.: No. pat. abnormal NoMatch records (180 d)	
Meds: No. Other pat. Records		Meds: No. ATC A pat. records	Medication in previous 120-180 days - ATC level 1 hierarchy
Meds: No. Outpatient pat. Records		Meds: No. ATC B pat. records	
Meds: No. Predadmission pat. Records		Meds: No. ATC C pat. records	
Meds: No. pat. GenericNameCode		Meds: No. ATC D pat. records	
Meds: No. pat. unique GenericNameCode		Meds: No. ATC G pat. records	
Meds: No. pat. BrandNameCode		Meds: No. ATC H pat. records	
Meds: No. pat. unique BrandNameCode		Meds: No. ATC J pat. records	
Meds: No. pat. unique Generic/BrandNameCode		Meds: No. ATC L pat. records	
		Meds: No. ATC M pat. records	
		Meds: No. ATC N pat. records	
		Meds: No. ATC P pat. records	
		Meds: No. ATC R pat. records	
		Meds: No. ATC S pat. records	
		Meds: No. ATC V pat. records	

* List of abbreviations is provided in Table 11

Supplementary Table S11 Abbreviations

VARIABLE NAME	ABBREVIATION
admission	adm.
Anatomical Therapeutic Chemical	ATC
emergency department	ED
Emergency	Emerg.
inpatient	inpat.
Index of Relative Socio-economic Advantage and Disadvantage	IRSAD
Medical	Med.
number	no.
patient	pat.
pathology	path.
previous	prev.
Private medical practitioner (not Psychiatrist)	Pvt. med. practit. (not Psychr.)
Radiology	Rad.
readmission	readm.
referral	ref.
rehabilitation	rehab.
Residential	Resid.
Socio-Economic Indexes for Areas	SEIFA
Surgical	Surg.

The total number of features used for modelling across all three hospital peer groups and outcome metrics are presented in Table S12 below.

Supplementary Table S12 Counts of candidate predictors across the hospital peer groups and outcome metrics

OUTCOME METRIC	COHORT	ALL+ DRG	ALL	MEDS	PATHO	BASIC
**RA30**	Children's hospital	168	73	61	58	46
	Principal referral	212	117	105	102	90
	Public acute	212	117	105	102	90
**RP 30**	Children's hospital	168	73	61	58	46
	Principal referral	212	117	105	102	90
	Public acute	212	117	105	102	90
**RP30E**	Children's hospital	117	97	66	61	30
	Principal referral	117	97	66	61	30
	Public acute	117	97	66	61	30

Appendix C Feature Implementation

This section describes the implementation of predictor variables in the final models. Due to data sparsity, categories in categorical variables may be collapsed. These are indicated with a mapping table between the original and collapsed categories.

Prev. inpat. stay count (for RA30, RP30)

Related variable: qhapdc_staycount_prev_1_180d (for RA30, RP30)

For a given admission with admission calendar date (D), counts the number of inpatient stays with a discharge day within 180 days (inclusive) of D. Excludes current stay.

Range: 0,1,2,…

Related quantities
qhapdc_staycount_prev_1_180d_sq= qhapdc_staycount_prev_1_180d^2
qhapdc_staycount_prev_1_180d_cu= qhapdc_staycount_prev_1_180d^3
qhapdc_staycount_prev_1_180d_quart= qhapdc_staycount_prev_1_180d^4
sqrt_qhapdc_staycount_prev_1_180d= sqrt(qhapdc_staycount_prev_1_180d)
log_qhapdc_staycount_prev_1_180d= log(1+qhapdc_staycount_prev_1_180d)

Analogous versions based on 120 days (120d) are defined similarly.

Prev. inpat. stay count (for RP30E)
Related variable: qhapdc_staycount_prev_1_180d (for RP30E)
For a given ED presentation with presentation calendar date (D), counts the number of inpatient stays with an admission in in the period {D-180,D-119,...,D-1}.

Range: 0,1,2,…

Related quantities
qhapdc_staycount_prev_1_180d_sq= qhapdc_staycount_prev_1_180d^2
qhapdc_staycount_prev_1_180d_cu= qhapdc_staycount_prev_1_180d^3
qhapdc_staycount_prev_1_180d_quart= qhapdc_staycount_prev_1_180d^4
sqrt_qhapdc_staycount_prev_1_180d= sqrt(qhapdc_staycount_prev_1_180d)

Analogous versions based on 120 days (120d) are defined similarly.

Prev. ED stay count
Related variable: ED_presentations_qhapdc_stays_prev_1_180d_counts
For a given admission with an admission calendar date (D), the number of ED presentations where the presentation day was in the period {D-180,D-119,...,D-1}

Range: 0,1,2,…

Related quantities
ED_presentations_qhapdc_stays_prev_1_180d_counts_sq=
ED_presentations_qhapdc_stays_prev_1_180d_counts^2
ED_presentations_qhapdc_stays_prev_1_180d_counts_cu=
ED_presentations_qhapdc_stays_prev_1_180d_counts^3
sqrt_ED_presentations_qhapdc_stays_prev_1_180d_counts=
sqrt(ED_presentations_qhapdc_stays_prev_1_180d_counts)
log_ED_presentations_qhapdc_stays_prev_1_180d_counts=
log(1+ED_presentations_qhapdc_stays_prev_1_180d_counts)

Analogous versions based on 120 days (120d) are defined similarly.

Prev. ED count
Related variable: ED_presentations_edcedis_stays_prev_1_180d_counts
For a given ED presentation with an a presentation date (D), the number of ED presentations where the presentation day was the in the period {D-180,D-119,...,D-1}.

Range: 0,1,2,…

Related quantities
ED_presentations_edcedis_stays_prev_1_180d_counts_sq=
ED_presentations_edcedis_stays_prev_1_180d_counts^2
ED_presentations_edcedis_stays_prev_1_180d_counts_cu=
ED_presentations_edcedis_stays_prev_1_180d_counts^3

Analogous versions based on 120 days (120d) are defined similarly.

Prev. inpat. stay count
Related variable: qhapdc_LOS_prev_1_180d
The sum of the length of stay (LOS) for all inpatient stays in the previous 180 days. Length of stay is defined as the difference between discharge and admission, with partial days expressed with fractional values.

Related quantities
qhapdc_LOS_prev_1_180d_sq, qhapdc_LOS_prev_1_180d_sqrt analogous to staycount calculations above.

Analogous versions based on 120 days (120d) are defined similarly.

Sex
Patient sex (Male or Female); patients with more than one sex code listed across their episodes are excluded due to data linkage concerns

Marital status
Possible values: Never married, Divorced, Married/de facto, Separated, Widowed Not stated/unknown

Age
Age is calculated as a decimal number in years as the number of weeks between the admission/presentation date and the patient's birth date (month and year) divided by 52.25. Newborn ages calculated as negative by this approach are set to 0.

Related quantities
age_sq=age^2
age_cu=age^3
age_quart=age^4

Indigenous status
Possible values: Indigenous, Not indigenous or unknown

Senior flag
A 0/1 binary indicator which is 1 for a stay if the patient's age for the stay is at least 65, and0 otherwise.

IRSAD decile
The Australian Bureau of Statistics (ABS) Socio-Economic Indexes for Areas (SEIFA) Index of Relative Socio-Economic Advantage and Disadvantage (IRSAD) value of SEIFA provided in the "seifa" variable of the QHAPDC and EDC data. "Unknown" or missing values are mapped to an "Unknown" category.
Possible values: 1,2,3,4,5,6,7,8,9,10,Unknown

Total prev. length of stay
Related variable: LOS
For inpatient stays, the number of nights between admission and discharge. Will be zero for episodes that start and end on the same day.

Related quantities
LOS_nights_sq= LOS_nights ^2
LOS_nights_cu= LOS_nights ^3

Sin/Cos of week number of discharge
The end date of an inpatient or ED stay, as the day of the (range 1—366), computed using the strftime() function and the "%j" format string. Useful for fitting temporal trends (e.g., seasonality).

Related quantities
disch_weekno=disch_dayno %/% 7
disch_weekno_sin=sin(2 * pi/52.17857 * (disch_weekno))
disch_weekno_cos=cos(2 * pi/52.17857 * (disch_weekno))
disch_weekno_sin_senior= disch_weekno_sin*senior_flag
disch_weekno_cos_senior= disch_weekno_cos*senior_flag

Adm source collapse

Related quantity: Adm source

Supplementary Table S13 Admission Source Mapping Table (Principal Referral and Public Acute Hospitals)

ADM SOURCE	ADM SOURCE
emergency department - this hospital	emergency department - this hospital
outpatient department - this hospital	outpatient department - this hospital
routine readmission not requiring referral	routine readmission not requiring referral
Admitted patient transferred from another hospital	Admitted patient transferred from another hospital
private medical practitioner (not psychiatrist)	private medical practitioner (not psychiatrist)
born in facility	born in facility
Other	other_collapsed
residential aged care service	residential aged care service
boarder	boarder
Non-Admitted patient referred from another hospital	Non-Admitted patient referred from another hospital
Community service	other_collapsed
episode change	other_collapsed
Planned emergency	other_collapsed
other health care establishment	other_collapsed
organ procurement	other_collapsed
private psychiatrist	other_collapsed

Adm source collapse

Related quantity: Adm_source

Supplementary Table S14 Admission Source Mapping Table (Children's Hospital)

ADM_SOURCE	ADM_SOURCE_COLLAPSE_CHILDRENS
emergency department - this hospital	emergency department - this hospital
outpatient department - this hospital	outpatient department - this hospital
routine readmission not requiring referral	other_collapsed
Admitted patient transferred from another hospital	Admitted patient transferred from another hospital
private medical practitioner (not psychiatrist)	private medical practitioner (not psychiatrist)
born in facility	other_collapsed
Other	other_collapsed
residential aged care service	other_collapsed
boarder	boarder
Non-Admitted patient referred from another hospital	other_collapsed
Community service	other_collapsed
episode change	other_collapsed
Planned emergency	other_collapsed
other health care establishment	other_collapsed
organ procurement	other_collapsed
private psychiatrist	other_collapsed

Adm unit

Related quantity: Adm_unit

Supplementary Table S15 Admission Unit Mapping Table

ADM_UNIT	ADM_UNIT_DESC	ADM_UNIT_COLLAPSE_4
ADDU	Alcohol & Drug Dependency	ADDU
CARD	Cardiology	CARD
CARS	Cardiac Surgical	CARS_CART
CART	Cardio Thoracic Surgical	CARS_CART
CCU	Coronary Care Unit	CCU
EMED	Emergency Medicine	EMED
ENDO	Endocrinology and Diabetes	ENDO
ENT	Ear Nose and Throat	ENT_MAXI
MAXI	Maxillofacial	ENT_MAXI
GAST	Gastroenterology	GAST
GENM	General Medical	GENM_GENO
GENO	General Medical/Surgical	GENM_GENO
GENS	General Surgical	GENS
HAEM	Haematology	HAEM
ICU	Intensive Care	ICU
IMMU	Immunology	IMMU_RHEU
RHEU	Rheumatology	IMMU_RHEU
NEUR	Neurology	NEUR
NSUR	Neurosurgery	NSUR
BIRT	Birthing Unit	OB_Birth_Maternity
OBST	Obstetric/Maternity	OB_Birth_Maternity
GYNA	Gynaecology Medical	OB_GYN_all
GYNC	Gynaecology Medical/Surgical	OB_GYN_all
GYNS	Gynaecology Surgical	OB_GYN_all
OBSG	Obstetrics/Gynaecology	OB_GYN_all
GYNO	Gynaecology Oncology	Oncol_MedSurgRad
ONCM	Oncology - Medical	Oncol_MedSurgRad
ONCS	Oncology - Surgical	Oncol_MedSurgRad
ONCR	Oncology - Radiation	Oncol_MedSurgRad
OPHT	Ophthalmology	OPHT
ORTH	Orthopaedic	ORTH
HIGH	High Dependency Unit	Other
HIV	AIDS/HIV Unit	Other
NEOI	Neonatal Intensive Care	Other
DENT	Dental/Oral	Other
INFE	Infectious Diseases	Other
DAY	Day Surgery	Other
DERM	Dermatology	Other
ENDS	Endoscopy	Other
GERI	Geriatric	Other
HYPE	Hyperbaric	Other
RADI	Radiology	Other

ADM_UNIT	ADM_UNIT_DESC	ADM_UNIT_COLLAPSE_4
SPIN	Spinal Injuries	Other
PAIN	PAIN	Other
ANAE	Anaesthetic/Pain Management	Other
OUTS	Outpatients - Specialists	Other
RESP	Respite	Other
SLEP	Sleep Centre	Other
WOCH	Women and Child Health Medical/Surgical	Other
PEEN	Paediatric - Ear Nose and Throat	PAED-ENT
NEON	Neonatology	PAED-General
PEGE	Paediatric - General	PAED-General
PEDE	Paediatric - Dental/Oral	PAED-OtherInternalExternal
PEDR	Paediatric - Dermatology	PAED-OtherInternalExternal
PEDV	Paediatric - Developmental	PAED-OtherInternalExternal
PEMA	Paediatric - Maxillofacial	PAED-OtherInternalExternal
PEOP	Paediatric - Ophthalmology	PAED-OtherInternalExternal
PEOR	Paediatric - Orthopaedic	PAED-OtherInternalExternal
PEPL	Paediatric - Plastic/Reconstructive Surgery/Burns	PAED-OtherInternalExternal
PECA	Paediatric - Cardiology	PAED-OtherInternalExternal
PECS	Paediatric - Cardiac Surgical	PAED-OtherInternalExternal
PECY	Paediatric - Cystic Fibrosis	PAED-OtherInternalExternal
PEED	Paediatric - Endocrinology/Diabetics	PAED-OtherInternalExternal
PEGA	Paediatric - Gastroenterology	PAED-OtherInternalExternal
PENE	Paediatric - Nephrology	PAED-OtherInternalExternal
PENL	Paediatric - Neurology	PAED-OtherInternalExternal
PERE	Paediatric - Respiratory Medicine	PAED-OtherInternalExternal
PERH	Paediatric - Rheumatology	PAED-OtherInternalExternal
PESP	Paediatric - Spinal	PAED-OtherInternalExternal
PEUR	Paediatric - Urology	PAED-OtherInternalExternal
PALG	Palliative - General	PALL-All
PALL	Palliative - Designated Unit	PALL-All
PEON	Paediatric - Oncology	PEON
PENS	Paediatric - Neurosurgery	PICU-PESU-PENS-PELT
PEIC	Paediatric - Intensive Care	PICU-PESU-PENS-PELT
PELT	Paediatric - Liver Transplant	PICU-PESU-PENS-PELT
PESU	Paediatric - Surgery	PICU-PESU-PENS-PELT
PLAS	Plastic/Reconstructive Surgery/Burns	PLAS
PYAA	Psychiatric Adult Acute Unit	PSYCH_Adult
PYAQ	Psychiatric Adult Ext - Acquired Brain Damage Unit	PSYCH_Adult
PYAW	Psychiatric Adult Special Care Suite	PSYCH_Adult
PYDD	Psychiatric Adult Extended - Dual Diagnosis Unit	PSYCH_Adult
PYET	Psychiatric Adult Extended - Treatment Rehab Unit	PSYCH_Adult
PYFA	Psychiatric Forensic Acute	PSYCH_Adult
PYGE	Psychiatric Older Persons -Acute	PSYCH_Adult
PYPG	Psychiatric Adult Extended - Psychogeriatric Unit	PSYCH_Adult
PYRA	Psychiatric Adult Residential	PSYCH_Adult
PYSH	Psychiatric Adult Ext - Extended High Security Unit	PSYCH_Adult
PYSM	Psychiatric Adult Ext - Extended Secure Medium Unit	PSYCH_Adult
PYYW	Psychiatric Adolescent Acute Unit in Adult Ward	PSYCH_Adult
PYOA	Psychiatric Young Persons (Youth) Acute Unit	PSYCH_Child_Adolescent
PYYA	Psychiatric Young Persons (Youth) Acute Unit	PSYCH_Child_Adolescent
PYCA	Psychiatric Child Acute Unit	PSYCH_Child_Adolescent
PYCW	Psychiatric Child Acute Unit in Paediatric Ward	PSYCH_Child_Adolescent
REHA	Rehabilitation -Geriatric	Rehab-All
REHD	Rehabilitation -Designated Unit	Rehab-All
REHG	Rehabilitation -General	Rehab-All
RENA	Renal/Nephrology	RENA
THOR	Thoracic/Respiratory Medical	Throacic-MedSurg
THOS	Thoracic Surgery	Throacic-MedSurg
TRAB	Transplantation Unit -Bone	Transplant-All
TRAH	Transplantation Unit -Heart/Lung	Transplant-All
TRAL	Transplantation Unit -Liver	Transplant-All
TRAM	Transplantation Unit -Bone Marrow	Transplant-All
TRAP	Transplantation Unit -Pancreas	Transplant-All
TRAR	Transplantation Unit -Renal	Transplant-All
UROL	Urology	UROL
VASC	Vascular	VASC
PYAM	Psychiatric Adult	PSYCH_Adult

Care type

Supplementary Table S16 Care type Mapping Table

CARE_TYPE	CARE_TYPE_COLLAPSE	CARE_TYPE_COLLAPSE_2
Acute	Acute	Acute
Mental Health	Mental Health	Mental Health
Newborn	Newborn	Newborn
Palliative care	Palliative	Palliative
Boarder	Other care	Other care
Rehabilitation care	Rehabilitation	Other care
Palliative - according to a designated program	Palliative	Palliative
Geriatric Evaluation and Management	Other care	Other care
Maintenance	Other care	Other care
Palliative - delivered in a designated unit	Palliative	Palliative
Psychogeriatric	Other care	Other care
Palliative - principal clinical intent	Palliative	Palliative
Other care	Other care	Other care
Rehabilitation - principal clinical intent	Rehabilitation	Other care
Organ procurement	Other care	Other care

Routine dialysis

For inpatient records, a 0/1 indicator where 1 indicates at least one patient record in the 120 days before the index stay is an episode that was not combined with others to make a larger stay, and the DRG 7.0 code is either "L61Z" (haemodialysis) or "L68Z" peritoneal dialysis.

The quantity routine_dialysis_episode_flag_prev_120_180d is analogous, for 120 to 180 days before the index stay.

DRG_70_category_{ZZZ}

Binary 0/1 indicators for the occurrence of a DRG 7.0 code with first character {ZZZ} in the 120 days before the index admission, where {ZZZ} can be one of the codes8 or A—S.

The quantities prev_120_180d_DRG_70_category_{ZZZ} are defined similarly for the period 120 to 180 days before the index admission.

Appendix D Demographic summary by dataset and readmission/representation status

Tables S17 – S19 provide an analytic summary of demographic data for RA30, RP30 and RP30E outcome metrics on training and test partitions of the data.

Supplementary Table S17 Demographic summary for RA30, by modelling dataset and representation status

	TRAIN		TEST	
	NO READMISSION	READMISSION	NO READMISSION	READMISSION
	(N=1,981,532)	(N=599,771)	(N=636,860)	(N=196,467)
Gender				
Female	1010301 (51.0%)	286205 (47.7%)	332892 (52.3%)	95770 (48.7%)
Male	971231 (49.0%)	313566 (52.3%)	303968 (47.7%)	100697 (51.3%)
Age				
Mean (SD)	48.2 (26.0)	52.6 (24.7)	49.3 (25.3)	53.9 (24.2)
Median (Min, Max)	51.3 [0, 118]	56.9 [0, 107]	52.1 [0, 119]	58.2 [0, 106]
Marital status				
Never married	709075 (35.8%)	197828 (33.0%)	220479 (34.6%)	62172 (31.6%)
Divorced	157835 (8.0%)	58971 (9.8%)	52433 (8.2%)	20188 (10.3%)
Married/de facto	863990 (43.6%)	258440 (43.1%)	284667 (44.7%)	86083 (43.8%)
Separated	70540 (3.6%)	24525 (4.1%)	23795 (3.7%)	8588 (4.4%)
Widowed	155594 (7.9%)	54443 (9.1%)	48288 (7.6%)	17684 (9.0%)
Not stated/unknown	24498 (1.2%)	5564 (0.9%)	7198 (1.1%)	1752 (0.9%)
Country of birth				
Australia	1525545 (77.0%)	459537 (76.6%)	486652 (76.4%)	149467 (76.1%)
Americas	18019 (0.9%)	4869 (0.8%)	6153 (1.0%)	1739 (0.9%)
At sea	4 (0%)	1 (0%)	0 (0%)	0 (0%)
New Zealand	100318 (5.1%)	30444 (5.1%)	33660 (5.3%)	10326 (5.3%)
North-East Asia	14978 (0.8%)	3308 (0.6%)	5339 (0.8%)	1520 (0.8%)
North-West Europe	162913 (8.2%)	54844 (9.1%)	51902 (8.1%)	17578 (8.9%)
North Africa and the Middle East	13048 (0.7%)	3611 (0.6%)	4563 (0.7%)	1231 (0.6%)
Oceania and Antarctica	23978 (1.2%)	7773 (1.3%)	7784 (1.2%)	2674 (1.4%)
South-East Asia	29865 (1.5%)	8327 (1.4%)	10355 (1.6%)	2932 (1.5%)
Southern and Central Asia	21357 (1.1%)	4860 (0.8%)	7693 (1.2%)	1801 (0.9%)
Southern and Eastern Europe	40400 (2.0%)	14083 (2.3%)	12544 (2.0%)	4461 (2.3%)
Sub-Saharan Africa	22117 (1.1%)	6400 (1.1%)	8003 (1.3%)	2295 (1.2%)
Not stated	8990 (0.5%)	1714 (0.3%)	2212 (0.3%)	443 (0.2%)
IRSAD decile				
1	288281 (14.5%)	90411 (15.1%)	93150 (14.6%)	30694 (15.6%)
2	237568 (12.0%)	70624 (11.8%)	71095 (11.2%)	21571 (11.0%)
3	226169 (11.4%)	69502 (11.6%)	76393 (12.0%)	24089 (12.3%)
4	195460 (9.9%)	58995 (9.8%)	62567 (9.8%)	19285 (9.8%)
5	191024 (9.6%)	55596 (9.3%)	65368 (10.3%)	18795 (9.6%)
6	185622 (9.4%)	54584 (9.1%)	62188 (9.8%)	18076 (9.2%)
7	186230 (9.4%)	56708 (9.5%)	58812 (9.2%)	17707 (9.0%)
8	173812 (8.8%)	53085 (8.9%)	57243 (9.0%)	17485 (8.9%)
9	146023 (7.4%)	45011 (7.5%)	43791 (6.9%)	14105 (7.2%)
10	101617 (5.1%)	34244 (5.7%)	30839 (4.8%)	10880 (5.5%)
Unknown	49726 (2.5%)	11011 (1.8%)	15414 (2.4%)	3780 (1.9%)
Indigenous status				
Indigenous	124336 (6.3%)	42517 (7.1%)	42883 (6.7%)	15149 (7.7%)
Not indigenous/unknown	1857196 (93.7%)	557254 (92.9%)	593977 (93.3%)	181318 (92.3%)
Hospital peer group				
Principal referral	763902 (38.6%)	268748 (44.8%)	262224 (41.2%)	92363 (47.0%)
Public acute groups	1139023 (57.5%)	300754 (50.1%)	351551 (55.2%)	96124 (48.9%)
Children's hospital	78607 (4.0%)	30269 (5.0%)	23085 (3.6%)	7980 (4.1%)
Inpatient stay count (180d)				
Mean (SD)	0.844 (2.32)	5.35 (12.0)	0.934 (2.43)	5.44 (12.2)
Median (Min, Max)	0 [0, 115]	2.00 [0, 194]	0 [0, 112]	2.00 [0, 149]
ED stay count (180d)				
Mean (SD)	0.817 (1.94)	2.41 (6.49)	0.853 (2.01)	2.37 (5.24)
Median (Min, Max)	0 [0, 142]	1.00 [0, 219]	0 [0, 134]	1.00 [0, 118]

Supplementary Table S18 Demographic summary for RP30, by modelling dataset and representation status

	TRAIN		TEST	
	NO REPRESENTATION	REPRESENTATION	NO REPRESENTATION	REPRESENTATION
	(N=3038575)	(N=697227)	(N=973970)	(N=229606)
Gender				
Female	1529539 (50.3%)	341980 (49.0%)	495104 (50.8%)	113694 (49.5%)
Male	1509036 (49.7%)	355247 (51.0%)	478866 (49.2%)	115912 (50.5%)
Age				
Mean (SD)	52.0 (24.0)	51.6 (24.4)	52.9 (23.2)	52.4 (24.2)
Median (Min, Max)	56.4 [0, 118]	55.0 [0, 109]	57.2 [0, 119]	56.1 [0, 108]
Marital status				
Never married	922849 (30.4%)	237496 (34.1%)	288882 (29.7%)	78192 (34.1%)
Divorced	274631 (9.0%)	67011 (9.6%)	90083 (9.2%)	22436 (9.8%)
Married/de facto	1448489 (47.7%)	294557 (42.2%)	472536 (48.5%)	96439 (42.0%)
Separated	118178 (3.9%)	30251 (4.3%)	38188 (3.9%)	10320 (4.5%)
Widowed	239293 (7.9%)	61783 (8.9%)	73726 (7.6%)	20471 (8.9%)
Not stated/unknown	35135 (1.2%)	6129 (0.9%)	10555 (1.1%)	1748 (0.8%)
Country of birth				
Australia	2301946 (75.8%)	544703 (78.1%)	731972 (75.2%)	179432 (78.1%)
Americas	27258 (0.9%)	4988 (0.7%)	9488 (1.0%)	1840 (0.8%)
At sea	5 (0%)	1 (0%)	0 (0%)	0 (0%)
New Zealand	158521 (5.2%)	32729 (4.7%)	53780 (5.5%)	11072 (4.8%)
North-East Asia	23197 (0.8%)	3713 (0.5%)	8974 (0.9%)	1311 (0.6%)
North-West Europe	246775 (8.1%)	55454 (8.0%)	78673 (8.1%)	17985 (7.8%)
North Africa and the Middle East	20533 (0.7%)	4400 (0.6%)	7309 (0.8%)	1422 (0.6%)
Oceania and Antarctica	62346 (2.1%)	12286 (1.8%)	19791 (2.0%)	4209 (1.8%)
South-East Asia	56700 (1.9%)	9473 (1.4%)	19340 (2.0%)	3175 (1.4%)
Southern and Central Asia	30834 (1.0%)	5609 (0.8%)	11097 (1.1%)	1954 (0.9%)
Southern and Eastern Europe	65086 (2.1%)	15660 (2.2%)	19128 (2.0%)	4648 (2.0%)
Sub-Saharan Africa	34043 (1.1%)	6671 (1.0%)	11818 (1.2%)	2185 (1.0%)
Not stated	11331 (0.4%)	1540 (0.2%)	2600 (0.3%)	373 (0.2%)
IRSAD decile				
1	466106 (15.3%)	117130 (16.8%)	150948 (15.5%)	39394 (17.2%)
2	381493 (12.6%)	88480 (12.7%)	113787 (11.7%)	27481 (12.0%)
3	352394 (11.6%)	82796 (11.9%)	119078 (12.2%)	29002 (12.6%)
4	306352 (10.1%)	70767 (10.1%)	98850 (10.1%)	24661 (10.7%)
5	298052 (9.8%)	67044 (9.6%)	98713 (10.1%)	22572 (9.8%)
6	280719 (9.2%)	60815 (8.7%)	93442 (9.6%)	21028 (9.2%)
7	277059 (9.1%)	61448 (8.8%)	85916 (8.8%)	18288 (8.0%)
8	255306 (8.4%)	57345 (8.2%)	84664 (8.7%)	18759 (8.2%)
9	212281 (7.0%)	48447 (6.9%)	63642 (6.5%)	14823 (6.5%)
10	151683 (5.0%)	31061 (4.5%)	46947 (4.8%)	9705 (4.2%)
Unknown	57130 (1.9%)	11894 (1.7%)	17983 (1.8%)	3893 (1.7%)
Indigenous status				
Indigenous	252453 (8.3%)	77897 (11.2%)	85304 (8.8%)	27320 (11.9%)
Not indigenous/unknown	2786122 (91.7%)	619330 (88.8%)	888666 (91.2%)	202286 (88.1%)
Hospital peer group				
Principal referral	1297866 (42.7%)	290824 (41.7%)	443908 (45.6%)	98277 (42.8%)
Public acute groups	1643769 (54.1%)	383970 (55.1%)	502596 (51.6%)	124667 (54.3%)
Children's hospital	96940 (3.2%)	22433 (3.2%)	27466 (2.8%)	6662 (2.9%)
Inpatient stay count (180d)				
Mean (SD)	13.8 (26.4)	12.4 (23.8)	13.6 (25.8)	13.0 (24.1)
Median (Min, Max)	1.00 [0, 154]	2.00 [0, 194]	1.00 [0, 152]	2.00 [0, 122]
ED stay count (180d)				
Mean (SD)	0.814 (1.51)	2.87 (6.83)	0.857 (1.56)	2.88 (5.64)
Median (Min, Max)	0 [0, 98.0]	1.00 [0, 219]	0 [0, 117]	

Supplementary Table S19 Demographic summary for RP30E, by modelling dataset and representation status

	TRAIN		TEST	
	NO REPRESENTATION	REPRESENTATION	NO REPRESENTATION	REPRESENTATION
	(N=2954135)	(N=964057)	(N=909992)	(N=308814)
Gender				
Female	1503671 (50.9%)	480939 (49.9%)	465289 (51.1%)	155021 (50.2%)
Male	1450464 (49.1%)	483118 (50.1%)	444703 (48.9%)	153793 (49.8%)
Age				
Mean (SD)	40.5 (26.4)	41.9 (25.1)	41.1 (26.6)	42.5 (25.4)
Median (Min, Max)	38.6 [0, 109]	40.0 [0, 109]	39.3 [0, 109]	40.9 [0, 106]
Country of birth				
Australia	2360251 (79.9%)	794992 (82.5%)	727794 (80.0%)	255334 (82.7%)
Americas	27238 (0.9%)	6979 (0.7%)	8627 (0.9%)	2429 (0.8%)
New Zealand	130322 (4.4%)	38928 (4.0%)	39401 (4.3%)	12176 (3.9%)
North-East Asia	23300 (0.8%)	4690 (0.5%)	7116 (0.8%)	1506 (0.5%)
North-West Europe	188542 (6.4%)	58906 (6.1%)	57231 (6.3%)	18527 (6.0%)
North Africa and the Middle East	20086 (0.7%)	5797 (0.6%)	6240 (0.7%)	1830 (0.6%)
Oceania and Antarctica	32832 (1.1%)	8956 (0.9%)	9990 (1.1%)	2936 (1.0%)
South-East Asia	38527 (1.3%)	8854 (0.9%)	12300 (1.4%)	2974 (1.0%)
Southern and Central Asia	34555 (1.2%)	8288 (0.9%)	11212 (1.2%)	2650 (0.9%)
Southern and Eastern Europe	46695 (1.6%)	14988 (1.6%)	13710 (1.5%)	4381 (1.4%)
Sub-Saharan Africa	32229 (1.1%)	8570 (0.9%)	10615 (1.2%)	2746 (0.9%)
Other	19558 (0.7%)	4109 (0.4%)	5756 (0.6%)	1325 (0.4%)
IRSAD decile				
1	421241 (14.3%)	158594 (16.5%)	134301 (14.8%)	53558 (17.3%)
2	407602 (13.8%)	152326 (15.8%)	103020 (11.3%)	38236 (12.4%)
3	313831 (10.6%)	111615 (11.6%)	114607 (12.6%)	44440 (14.4%)
4	247609 (8.4%)	77549 (8.0%)	74611 (8.2%)	24648 (8.0%)
5	275546 (9.3%)	85973 (8.9%)	98698 (10.8%)	32972 (10.7%)
6	271057 (9.2%)	77795 (8.1%)	86616 (9.5%)	25351 (8.2%)
7	280051 (9.5%)	83904 (8.7%)	78449 (8.6%)	23383 (7.6%)
8	268087 (9.1%)	79501 (8.2%)	83688 (9.2%)	25102 (8.1%)
9	223486 (7.6%)	65309 (6.8%)	64391 (7.1%)	18858 (6.1%)
10	165870 (5.6%)	44941 (4.7%)	48750 (5.4%)	13624 (4.4%)
Unknown	79755 (2.7%)	26550 (2.8%)	22861 (2.5%)	8642 (2.8%)
Indigenous status				
Indigenous	214207 (7.3%)	106240 (11.0%)	72552 (8.0%)	37334 (12.1%)
Not indigenous/unknown	2739928 (92.7%)	857817 (89.0%)	837440 (92.0%)	271480 (87.9%)
Hospital peer group				
Principal referral	923462 (31.3%)	293179 (30.4%)	307810 (33.8%)	98895 (32.0%)
Public acute groups	1899387 (64.3%)	637218 (66.1%)	562620 (61.8%)	199607 (64.6%)
Children’s hospital	131286 (4.4%)	33660 (3.5%)	39562 (4.3%)	10312 (3.3%)
Inpatient stay count (180d)				
Mean (SD)	0.737 (3.62)	2.13 (6.52)	0.804 (3.78)	2.26 (6.48)
Median (Min, Max)	0 [0, 145]	0 [0, 195]	0 [0, 115]	1.00 [0, 115]
ED stay count (180d)				
Mean (SD)	0.860 (1.71)	3.87 (8.84)	0.901 (1.77)	4.15 (9.07)
Median (Min, Max)	0 [0, 146]	1.00 [0, 219]	0 [0, 99.0]	1.00 [0, 140]

Appendix E Predictors included in the pruned final models

Once the best set of hyperparameters were selected for each model candidate and models retrained on the whole training data, we analysed feature importance for each model individually by computing the Shapley values for the whole training data. Features contributing the most towards high AUC performance were characterised as the most important. Analysis of the results implied that for every hospital peer group and every outcome metric, at least half of the features made a negligible contribution and as such were redundant. Also, it is interesting to note that the features of age, ED presentations, previous inpatient stay count, number of unique patient tests, stay count and Length of Stay (LOS) were systematically highest ranked across all hospital peer groups and outcome metrics indicating their global importance for predicting hospitalisation risk. It also explains why a model obtained using the data group denoted as the 'Basic' model had comparable performance to models comprising all types of features. Consequently, the obtained Shapley values are used as inputs into the second stage of modelling which aimed at removing features making negligible contributions from every model individually. Features comprising the pruned models for the three hospital peer groups are reported in Tables S20, S21 and S22. AUC performance presented in Table 1 of the main manuscript confirms how little is added to model discrimination by the eliminated features.

Supplementary Table S20 Children's hospitals: Features used in the final models across the outcome metrics

RA30	RP30	RP30E
Prev. inpat. stay count (180 d)	Prev. inpat. stay count (180 d)	Prev. inpat. stay count (180 d)
Prev. ED stay count (180 d)	Prev. ED stay count (180 d)	Prev. ED stay count (180 d)
Age	Age	Age
Path.: Pat. abnormal AllDep flag (180 d)	Path.: Pat. abnormal AllDep flag (180 d)	Path.: No. unique patient tests (180 d)
Path.: No. unique patient tests (180 d)	Path.: Pat. abnormal Immu flag (180 d)	Path.: No. unique patient specimens (180 d)
Path.: No. unique patient specimens (180 d)	Path.: No. unique patient tests (180 d)	Path.: No. pat. observable codes (180 d)
Path.: No. pat. observable codes (180 d)	Path.: No. unique patient specimens (180 d)	Path.: No. pat. abnormal (LL, L, H, HH) codes (180 d)
Path.: No. pat. unique observable codes (180 d)	Path.: No. pat. observable codes (180 d)	Week no. of discharge
SEIFA IRSAD decile: 4	Sex: Male	Total prev. length of stay (180 d)
SEIFA IRSAD decile: 9	SEIFA IRSAD decile: 3	
Planned same day	SEIFA IRSAD decile: 10	
Care type: Acute	SEIFA IRSAD decile: Unknown	
Care type: Other care	Planned same day	
Elective status: Emerg. adm.	Care type: Acute	
Elective status: Not assigned	Care type: Other care	
Adm. source collapse (Childrens): Admitted transfer in	Elective status: Emerg. adm.	
Adm. source collapse (Childrens): Boarder	Elective status: Not assigned	
Adm. source collapse (Childrens): ED-this hospital	Adm. source collapse (Childrens): ED-this hospital	
Adm. source collapse (Childrens): Outpatient-this hospital	Week no. of discharge	
Adm. source collapse (Childrens): Pvt. med. practit. (not Psychr.)	Total prev. length of stay (180 d)	
Week no. of discharge		
Cosine of week no. of discharge		
Total prev. length of stay (180 d)		

* List of abbreviations is provided in Table 8

Supplementary Table S21 Principal referral hospitals: Features used in the final models across the outcome metrics

RA30	RP30	RP30E
Prev. inpat. stay count (180 d)	Prev. inpat. stay count (180 d)	Prev. inpat. stay count (180 d)
Prev. ED stay count (180 d)	Prev. ED stay count (180 d)	Prev. ED stay count (180 d)
Age	Age	Age
Age^2	Age^2	Path.: No. unique patient tests (180 d)
Path.: Pat. abnormal AllDep flag (180 d)	Path.: Pat. abnormal AllDep flag (180 d)	Path.: No. unique patient specimens (180 d)
Path.: Pat. abnormal Chempath. flag (180 d)	Path.: Pat. abnormal Chempath. flag (180 d)	Path.: No. pat. unique observable codes (180 d)
Path.: Pat. abnormal Cytology flag (180 d)	Path.: Pat. abnormal Cytology flag (180 d)	Path.: No. pat. abnormal L codes (180 d)
Path.: Pat. abnormal Haemotology flag (180 d)	Path.: Pat. abnormal Haemotology flag (180 d)	Path.: No. pat. abnormal H codes (180 d)
Path.: Pat. abnormal Immu flag (180 d)	Path.: Pat. abnormal Immu flag (180 d)	Path.: No. pat. abnormal HH codes (180 d)
Path.: Pat. abnormal Sero flag (180 d)	Path.: Pat. abnormal Sero flag (180 d)	Path.: No. pat. abnormal (LL, L, H, HH) codes (180 d)
Path.: No. unique patient tests (180 d)	Path.: Pat. abnormal Trans flag (180 d)	Path.: No. pat. abnormal Chempath. records (180 d)
Path.: No. unique patient specimens (180 d)	Path.: No. unique patient tests (180 d)	Path.: No. pat. abnormal Haematology records (180 d)
Path.: No. pat. observable codes (180 d)	Path.: No. unique patient specimens (180 d)	Path.: No. pat. abnormal Immu records (180 d)
Path.: No. pat. unique observable codes (180 d)	Path.: No. pat. observable codes (180 d)	Path.: No. pat. abnormal Sero records (180 d)
Sex: Male	Path.: No. pat. unique observable codes (180 d)	Sex: Male
Marital status: Married/de facto	Sex: Male	Indigenous status: Indigenous
Marital status: Not stated/unknown	Marital status: Divorced	SEIFA IRSAD decile: 3
Indigenous status: Indigenous	Marital status: Married/de facto	SEIFA IRSAD decile: 10
SEIFA IRSAD decile: 2	Marital status: Not stated/unknown	SEIFA IRSAD decile: Unknown
SEIFA IRSAD decile: 3	Marital status: Separated	Week no. of discharge
SEIFA IRSAD decile: 4	Marital status: Widowed	Cosine of week no. of discharge
SEIFA IRSAD decile: 6	Indigenous status: Indigenous	Total prev. length of stay (180 d)
SEIFA IRSAD decile: 7	SEIFA IRSAD decile: 1	
SEIFA IRSAD decile: 9	SEIFA IRSAD decile: 2	
SEIFA IRSAD decile: 10	SEIFA IRSAD decile: 3	
SEIFA IRSAD decile: Unknown	SEIFA IRSAD decile: 4	
Adm. source: Outpatient-this hospital	SEIFA IRSAD decile: 5	
Adm. source: Pvt. med. practit. (not Psychr.)	SEIFA IRSAD decile: 7	
Adm. source: Resid. aged care	SEIFA IRSAD decile: 8	
Adm. source: Routine readm. not requiring ref.	SEIFA IRSAD decile: 9	
Planned same day	SEIFA IRSAD decile: 10	
Adm. unit: ADDU	SEIFA IRSAD decile: Unknown	
Adm. unit: CARD	Adm. source: Admitted transfer in	
Adm. unit: CARS, CART	Adm. source: Non-admitted referred	
Adm. unit: EMED	Adm. source: Boarder	
Adm. unit: ENT, MAXI	Adm. source: Outpatient-this hospital	
Adm. unit: GAST	Adm. source: Pvt. med. practit. (not Psychr.)	
Adm. unit: GENM, GENO	Adm. source: Routine readm. not requiring ref.	
Adm. unit: GENS	Planned same day	
Adm. unit: HAEM	Adm. unit: CARD	
Adm. unit: IMMU, RHEU	Adm. unit: EMED	
Adm. unit: NEUR	Adm. unit: ENT, MAXI	
Adm. unit: NSUR	Adm. unit: GAST	
Adm. unit: OB (Birth/Maternity)	Adm. unit: GENM, GENO	
Adm. unit: all other (OB/GYN)	Adm. unit: GENS	
Adm. unit: OPHT	Adm. unit: HAEM	
Adm. unit: ORTH	Adm. unit: NEUR	
Adm. unit: Oncology (Med., Surg., Rad.)	Adm. unit: OB (Birth/Maternity)	
Adm. unit: Other	Adm. unit: all other (OB/GYN)	
Adm. unit: PAED (General)	Adm. unit: OPHT	
Adm. unit: PICU, PESU, PENS, PELT	Adm. unit: ORTH	
Adm. unit: PLAS	Adm. unit: Oncology (Med., Surg., Rad.)	
Adm. unit: PSYCH (Adult)	Adm. unit: Other	
Adm. unit: RENA	Adm. unit: PAED (General)	
Adm. unit: all rehab.	Adm. unit: all palliative	
Adm. unit: Thoracic (Med., Surg.)	Adm. unit: RENA	
Adm. unit: all transplant	care type: Acute	
Adm. unit: UROL	care type: Mental Health	
care type: Acute	care type: Other care	
care type: Mental Health	Elective status: Emerg. adm.	
Elective status: Emerg. adm.	Elective status: Not assigned	
Elective status: Not assigned	Week no. of discharge	
Week no. of discharge	Sine of week no. of discharge	
Sine of week no. of discharge	Sine of week no. of discharge (senior only)	
Sine of week no. of discharge (senior only)	Cosine of week no. of discharge	
Cosine of week no. of discharge	Cosine of week no. of discharge (senior only)	
Cosine of week no. of discharge (senior only)	Total prev. length of stay (180 d)	
Total prev. length of stay (180 d)		

* List of abbreviations is provided in Table 8

Supplementary Table S22 Public acute hospitals: Features used in the final models across the outcome metrics

RA30	RP30	RP30E
Prev. inpat. stay count (180 d)	Prev. inpat. stay count (180 d)	Prev. inpat. stay count (180 d)
Prev. ED stay count (180 d)	Prev. ED stay count (180 d)	Prev. ED stay count (180 d)
Age	Age	Age
Path.: Pat. abnormal AllDep flag (180 d)	Age^3	Path.: No. unique patient tests (180 d)
Path.: Pat. abnormal Chempath. flag (180 d)	Path.: Pat. abnormal AllDep flag (180 d)	Path.: No. unique patient specimens (180 d)
Path.: Pat. abnormal Cytology flag (180 d)	Path.: Pat. abnormal Chempath. flag (180 d)	Path.: No. pat. unique observable codes (180 d)
Path.: Pat. abnormal Haemotology flag (180 d)	Path.: Pat. abnormal Cytology flag (180 d)	Path.: No. pat. abnormal L codes (180 d)
Path.: Pat. abnormal Sero flag (180 d)	Path.: Pat. abnormal Haemotology flag (180 d)	Path.: No. pat. abnormal H codes (180 d)
Path.: No. unique patient tests (180 d)	Path.: Pat. abnormal Immu flag (180 d)	Path.: No. pat. abnormal HH codes (180 d)
Path.: No. unique patient specimens (180 d)	Path.: Pat. abnormal Sero flag (180 d)	Path.: No. pat. abnormal (LL, L, H, HH) codes (180 d)
Path.: No. pat. observable codes (180 d)	Path.: No. unique patient tests (180 d)	Path.: No. pat. abnormal Chempath. records (180 d)
Path.: No. pat. unique observable codes (180 d)	Path.: No. unique patient specimens (180 d)	Path.: No. pat. abnormal Cytology records (180 d)
Sex: Male	Path.: No. pat. observable codes (180 d)	Path.: No. pat. abnormal Haematology records (180 d)
Marital status: Divorced	Path.: No. pat. unique observable codes (180 d)	Path.: No. pat. abnormal Sero records (180 d)
Marital status: Married/de facto	Sex: Male	Sex: Male
Marital status: Not stated/unknown	Marital status: Married/de facto	Indigenous status: Indigenous
Indigenous status: Indigenous	Marital status: Not stated/unknown	SEIFA IRSAD decile: 1
SEIFA IRSAD decile: 1	Marital status: Separated	SEIFA IRSAD decile: 2
SEIFA IRSAD decile: 2	Marital status: Widowed	SEIFA IRSAD decile: 3
SEIFA IRSAD decile: 9	Indigenous status: Indigenous	SEIFA IRSAD decile: 4
SEIFA IRSAD decile: 10	SEIFA IRSAD decile: 1	SEIFA IRSAD decile: 6
SEIFA IRSAD decile: Unknown	SEIFA IRSAD decile: 3	SEIFA IRSAD decile: 8
Adm. source: Admitted transfer in	SEIFA IRSAD decile: 4	SEIFA IRSAD decile: 9
Adm. source: Non-admitted referred	SEIFA IRSAD decile: 5	SEIFA IRSAD decile: 10
Adm. source: Outpatient-this hospital	SEIFA IRSAD decile: 7	SEIFA IRSAD decile: Unknown
Adm. source: Pvt. med. practit. (not Psychr.)	SEIFA IRSAD decile: 8	Week no. of discharge
Adm. source: Routine readm. not requiring ref.	SEIFA IRSAD decile: 9	Cosine of week no. of discharge
Planned same day	SEIFA IRSAD decile: 10	Total prev. length of stay (180 d)
Adm. unit: ADDU	SEIFA IRSAD decile: Unknown	
Adm. unit: CARD	Adm. source: Non-admitted referred	
Adm. unit: EMED	Adm. source: Boarder	
Adm. unit: ENT, MAXI	Adm. source: Outpatient-this hospital	
Adm. unit: GAST	Adm. source: Pvt. med. practit. (not Psychr.)	
Adm. unit: GENM, GENO	Adm. source: Routine readm. not requiring ref.	
Adm. unit: GENS	Planned same day	
Adm. unit: ICU	Adm. unit: CARD	
Adm. unit: OB (Birth/Maternity)	Adm. unit: EMED	
Adm. unit: all other (OB/GYN)	Adm. unit: ENT, MAXI	
Adm. unit: OPHT	Adm. unit: GAST	
Adm. unit: Other	Adm. unit: GENM, GENO	
Adm. unit: all palliative	Adm. unit: GENS	
Adm. unit: PSYCH (Adult)	Adm. unit: HAEM	
Adm. unit: PSYCH (Child/Adolescent)	Adm. unit: NEUR	
Adm. unit: UROL	Adm. unit: OB (Birth/Maternity)	
Care type: Acute	Adm. unit: OPHT	
Care type: Mental Health	Adm. unit: Oncology (Med., Surg., Rad.)	
Care type: Other care	Adm. unit: Other	
Elective status: Emerg. adm.	Adm. unit: PAED (General)	
Elective status: Not assigned	Adm. unit: all palliative	
Week no. of discharge	Adm. unit: RENA	
Sine of week no. of discharge	Care type: Mental Health	
Sine of week no. of discharge (senior only)	Care type: Other care	
Cosine of week no. of discharge	Elective status: Emerg. adm.	
Cosine of week no. of discharge (senior only)	Elective status: Not assigned	
Total prev. length of stay (180 d)	Week no. of discharge	
	Sine of week no. of discharge	
	Cosine of week no. of discharge	
	Cosine of week no. of discharge (senior only)	
	Total prev. length of stay (180 d)	

* List of abbreviations is provided in Table 8

Appendix F Hyperparameter tuning

Hyperparameter tuning was used to find the best set of hyperparameters for each method to maximise AUC. A grid search over proposed values was used, fitting models to the training data. The grid search was implemented with the *sklearn* package and *GridSearchCV* library from the *model_selection* module. The cross-validation strategy was specified to be stratified 3-fold cross validation with the *refit* argument set to *True* which refits the estimator using the parameters found to be best on the whole dataset. Stratified cross validation ensured that all folds contained the same distribution of classes. Values for the grid search were chosen to cover the range 0—1 (for the L1 regularisation) and by domain expertise. Details of the parameter grid for model candidates are presented in Table S23. Tables S24-26 list the final sets of hyperparameters across the hospital peer groups and considered outcome metrics (RA30, RP30 and RP30E).

Supplementary Table S23 Hyperparameters grid

HOSPITAL PEER GROUP	METHOD	GRID
Children's hospital	L1	C = ['1e-03', '2.15443469e-03', '5.99484250e-03', '1.66810054e-02', '4.64158883e-02', '1.29154967e-01', '3.59381366e-01','1.00000000e+00']
Principal referral hospitals, Public acute hospitals		C = ['1.00000000e-04', '2.78255940e-04', '7.74263683e-04', '2.15443469e-03', '5.99484250e-03', '1.66810054e-02', '4.64158883e-02', '1.29154967e-01', '1.00000000e+00']
Children's hospital, Principal referral hospitals, Public acute hospitals	XGB	max_depth: [6, 10, 20],<br>n_estimators: [50, 70, 100],<br>gamma: [0, 10, 20, 50, 1000],<br>objective: ['binary:logistic'],<br>eval_metric: ['auc']
Children's hospital, Principal referral hospitals, Public acute hospitals	RF	n_estimators = [30, 50, 100]<br>max_features = ['auto']<br>max_depth = [3, 5, 7, 10]<br>min_samples_split = [10]<br>min_samples_leaf = [10]<br>bootstrap = [True]<br>class_weight = [None]

Supplementary Table S24 Final sets of hyperparameters across the hospital peer groups and RA30 outcome metric for data group Patho and historical window 180 days

HOSPITAL PEER GROUP	PARAMETERS
Children's hospital	XGBClassifier(base_score=0.5, booster='gbtree', colsample_bylevel=1,<br>colsample_bynode=1, colsample_bytree=1, eval_metric='auc',<br>gamma=10, gpu_id=-1, importance_type='gain',<br>interaction_constraints='', learning_rate=0.300000012,<br>max_delta_step=0, max_depth=6, min_child_weight=1, missing=nan,<br>monotone_constraints='()', n_estimators=50, n_jobs=8,<br>num_parallel_tree=1, random_state=0, reg_alpha=0, reg_lambda=1,<br>scale_pos_weight=1, subsample=1, tree_method='exact',<br>validate_parameters=1, verbosity=None)
Principal referral	XGBClassifier(base_score=0.5, booster='gbtree', colsample_bylevel=1,<br>colsample_bynode=1, colsample_bytree=1, eval_metric='auc',<br>gamma=20, gpu_id=-1, importance_type='gain',<br>interaction_constraints='', learning_rate=0.300000012,<br>max_delta_step=0, max_depth=20, min_child_weight=1, missing=nan,<br>monotone_constraints='()', n_estimators=50, n_jobs=8,<br>num_parallel_tree=1, random_state=0, reg_alpha=0, reg_lambda=1,<br>scale_pos_weight=1, subsample=1, tree_method='exact',<br>validate_parameters=1, verbosity=None)
Public acute	XGBClassifier(base_score=0.5, booster='gbtree', colsample_bylevel=1,<br>colsample_bynode=1, colsample_bytree=1, eval_metric='auc',<br>gamma=20, gpu_id=-1, importance_type='gain',<br>interaction_constraints='', learning_rate=0.300000012,<br>max_delta_step=0, max_depth=20, min_child_weight=1, missing=nan,<br>monotone_constraints='()', n_estimators=50, n_jobs=8,<br>num_parallel_tree=1, random_state=0, reg_alpha=0, reg_lambda=1,<br>scale_pos_weight=1, subsample=1, tree_method='exact',<br>validate_parameters=1, verbosity=None)

Supplementary Table S25 Final sets of hyperparameters across the hospital peer groups and RP30 outcome metric for data group Patho and historical window 180 days

HOSPITAL PEER GROUP	PARAMETERS
Children's hospital	XGBClassifier(base_score=0.5, booster='gbtree', colsample_bylevel=1, colsample_bynode=1, colsample_bytree=1, eval_metric='auc', gamma=50, gpu_id=-1, importance_type='gain', interaction_constraints='', learning_rate=0.300000012, max_delta_step=0, max_depth=10, min_child_weight=1, missing=nan, monotone_constraints='()', n_estimators=50, n_jobs=8, num_parallel_tree=1, random_state=0, reg_alpha=0, reg_lambda=1, scale_pos_weight=1, subsample=1, tree_method='exact', validate_parameters=1, verbosity=None)
Principal referral	XGBClassifier(base_score=0.5, booster='gbtree', colsample_bylevel=1, colsample_bynode=1, colsample_bytree=1, eval_metric='auc', gamma=50, gpu_id=-1, importance_type='gain', interaction_constraints='', learning_rate=0.300000012, max_delta_step=0, max_depth=6, min_child_weight=1, missing=nan, monotone_constraints='()', n_estimators=50, n_jobs=8, num_parallel_tree=1, random_state=0, reg_alpha=0, reg_lambda=1, scale_pos_weight=1, subsample=1, tree_method='exact', validate_parameters=1, verbosity=None)
Public acute	XGBClassifier(base_score=0.5, booster='gbtree', colsample_bylevel=1, colsample_bynode=1, colsample_bytree=1, eval_metric='auc', gamma=50, gpu_id=-1, importance_type='gain', interaction_constraints='', learning_rate=0.300000012, max_delta_step=0, max_depth=6, min_child_weight=1, missing=nan, monotone_constraints='()', n_estimators=50, n_jobs=8, num_parallel_tree=1, random_state=0, reg_alpha=0, reg_lambda=1, scale_pos_weight=1, subsample=1, tree_method='exact', validate_parameters=1, verbosity=None)

Supplementary Table S26 Final sets of hyperparameters across the hospital peer groups and RP30E outcome metric for data group Patho and historical window 180 days

HOSPITAL PEER GROUP	PARAMETERS
Children's hospital	XGBClassifier(base_score=0.5, booster='gbtree', colsample_bylevel=1, colsample_bynode=1, colsample_bytree=1, eval_metric='auc', gamma=50, gpu_id=-1, importance_type='gain', interaction_constraints='', learning_rate=0.300000012, max_delta_step=0, max_depth=6, min_child_weight=1, missing=nan, monotone_constraints='()', n_estimators=50, n_jobs=8, num_parallel_tree=1, random_state=0, reg_alpha=0, reg_lambda=1, scale_pos_weight=1, subsample=1, tree_method='exact', validate_parameters=1, verbosity=None)
Principal referral	XGBClassifier(base_score=0.5, booster='gbtree', colsample_bylevel=1, colsample_bynode=1, colsample_bytree=1, eval_metric='auc', gamma=20, gpu_id=-1, importance_type='gain', interaction_constraints='', learning_rate=0.300000012, max_delta_step=0, max_depth=6, min_child_weight=1, missing=nan, monotone_constraints='()', n_estimators=50, n_jobs=8, num_parallel_tree=1, random_state=0, reg_alpha=0, reg_lambda=1, scale_pos_weight=1, subsample=1, tree_method='exact', validate_parameters=1, verbosity=None)
Public acute	XGBClassifier(base_score=0.5, booster='gbtree', colsample_bylevel=1, colsample_bynode=1, colsample_bytree=1, eval_metric='auc', gamma=20, gpu_id=-1, importance_type='gain', interaction_constraints='', learning_rate=0.300000012, max_delta_step=0, max_depth=6, min_child_weight=1, missing=nan, monotone_constraints='()', n_estimators=50, n_jobs=8, num_parallel_tree=1, random_state=0, reg_alpha=0, reg_lambda=1, scale_pos_weight=1, subsample=1, tree_method='exact', validate_parameters=1, verbosity=None)

Appendix G AUC summary results

Figure S1 summarise the AUC summary results obtained with the L1 model on test data for different data groups (Basic, Meds, Patho, All, All+ DRG, Expert Model), historical windows (120 and 180 days) and outcome metrics (RA30, RP30 and RP30E).

Figure S2 summarise the AUC summary results obtained with tree-based ensemble models (XGB and RF models) on test data for different data groups (Basic, Meds, Patho, All, All+ DRG, Expert Model), historical windows (120 and 180 days) and outcome metrics (RA30, RP30 and RP30E).

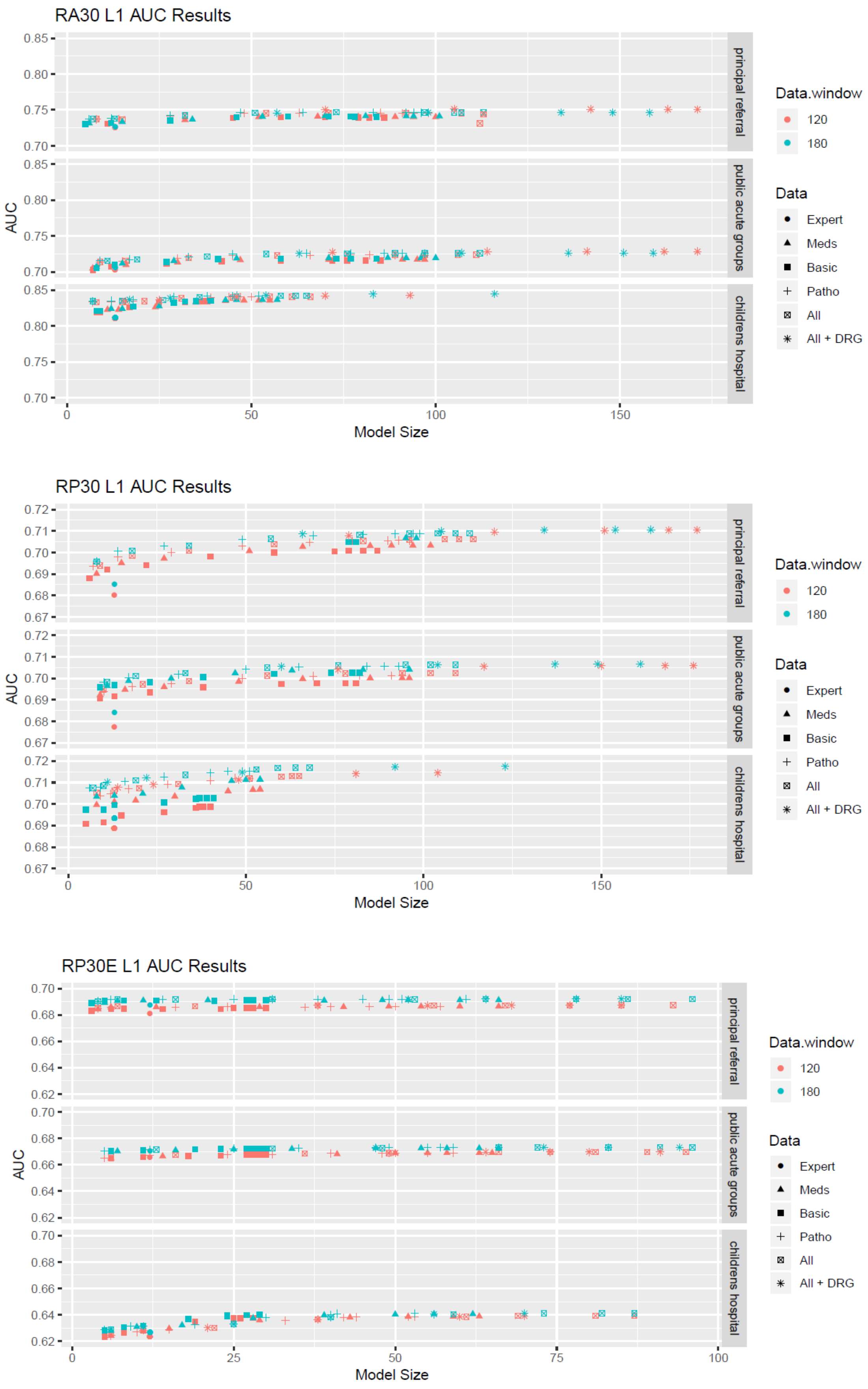

Supplementary Figure S1 L1 model: AUC as a function of model size across the cohorts and outcome metrics for different data groups

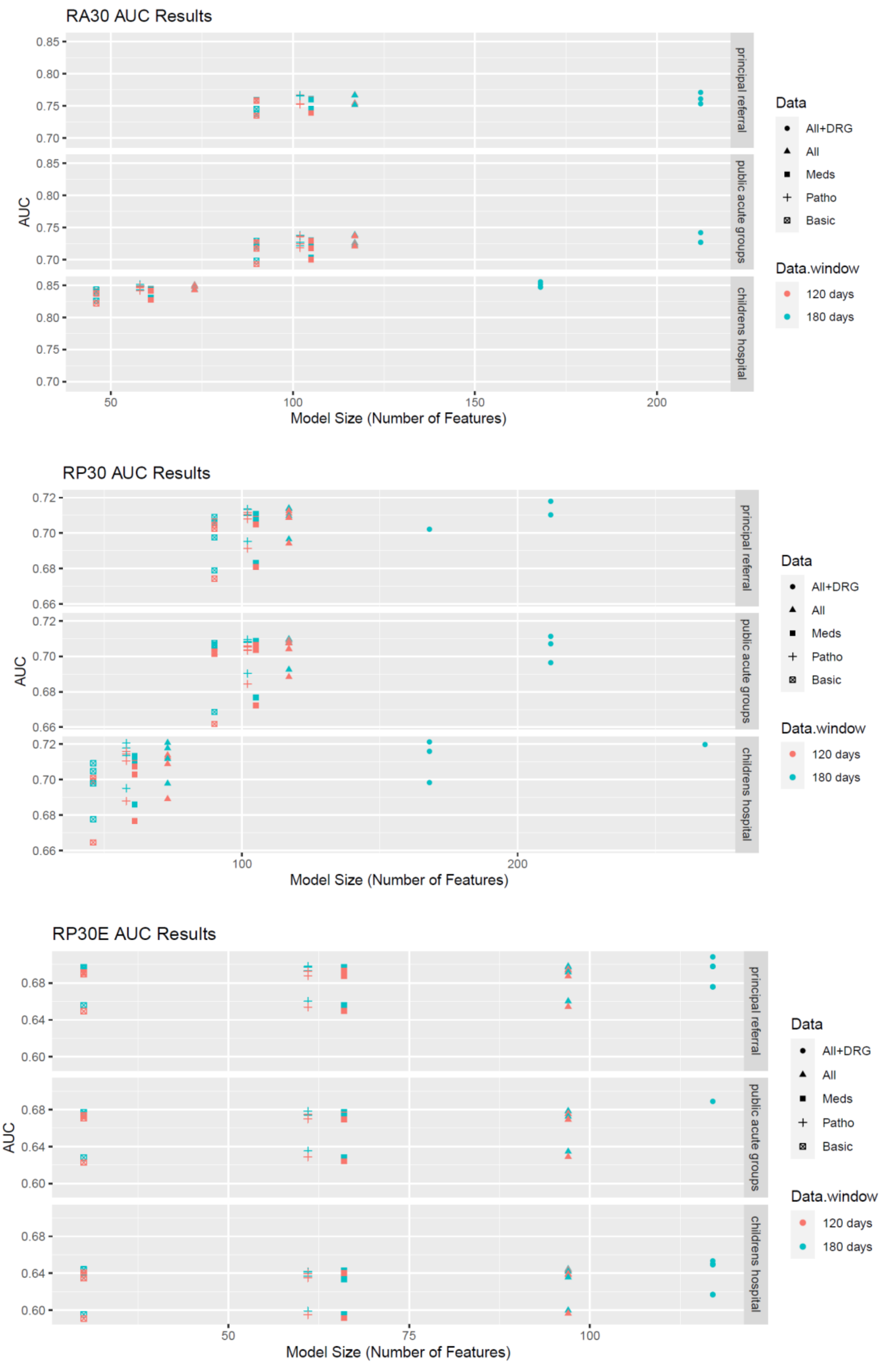

Supplementary Figure S2 RF and XGB models: AUC performance for different data groups across the cohorts and outcome metrics

Appendix H Explainability plots

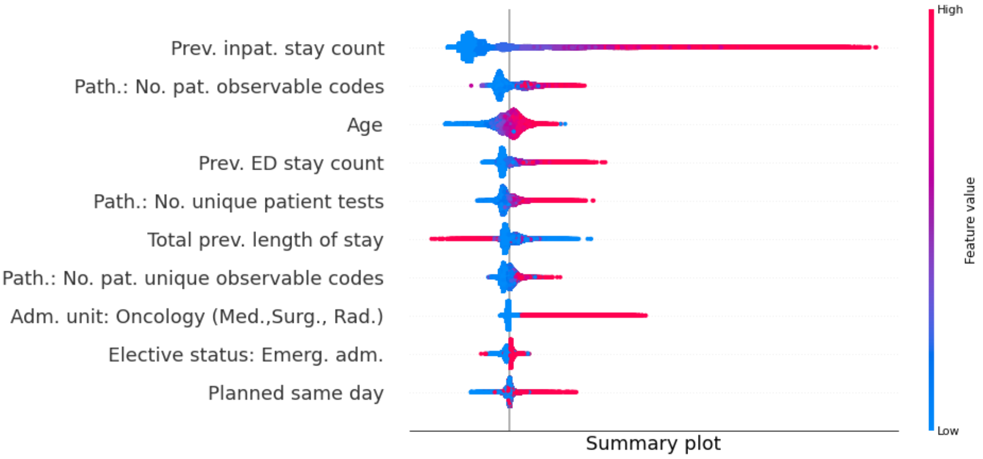

a) **RA30: Principal referral hospitals**

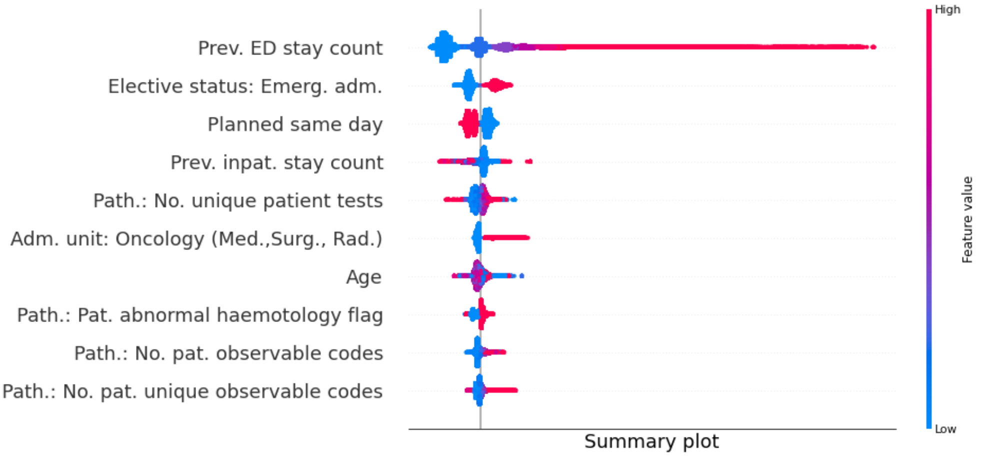

b) **RP30: Principal referral hospitals**

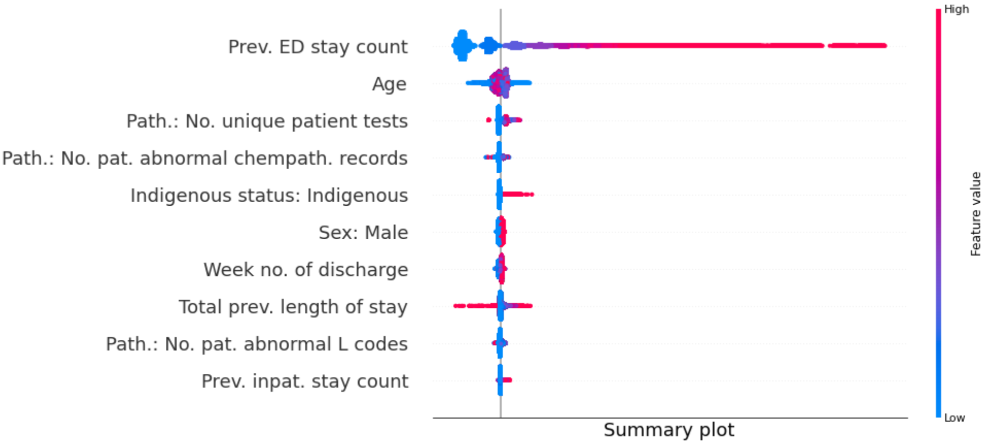

c) **RP30E: Principal referral hospitals**

Supplementary Figure S3 Principal referral hospitals: Summary plot of Shapley values computed for each patient individually in the test partition. Features are sorted top-down based on their global contribution. The distance of a dot representing a sample from the vertical line indicates its contribution. The colour of a dot indicates feature

value for that sample. Blue and pink colour represent extreme values of the feature. Shapley values on the right side of vertical axes 'push' predictions towards the class 1 and those on the left side towards the class 0.

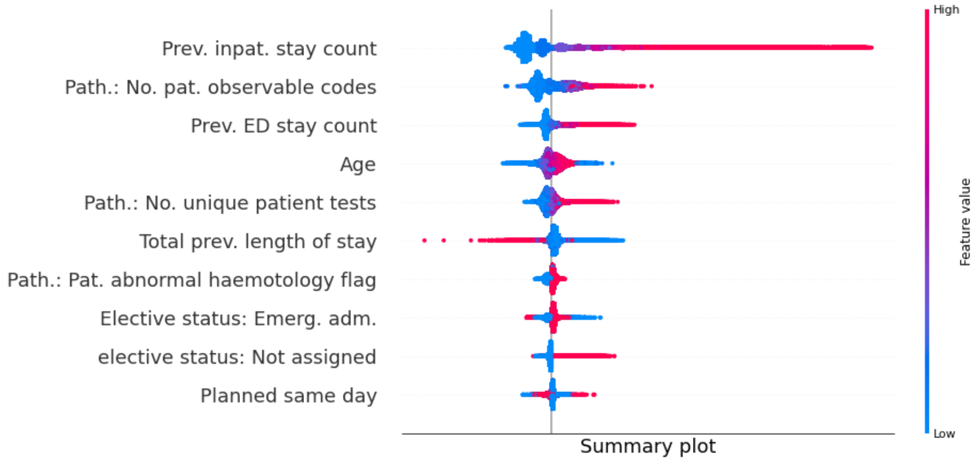

a) **RA30: Public acute hospitals**

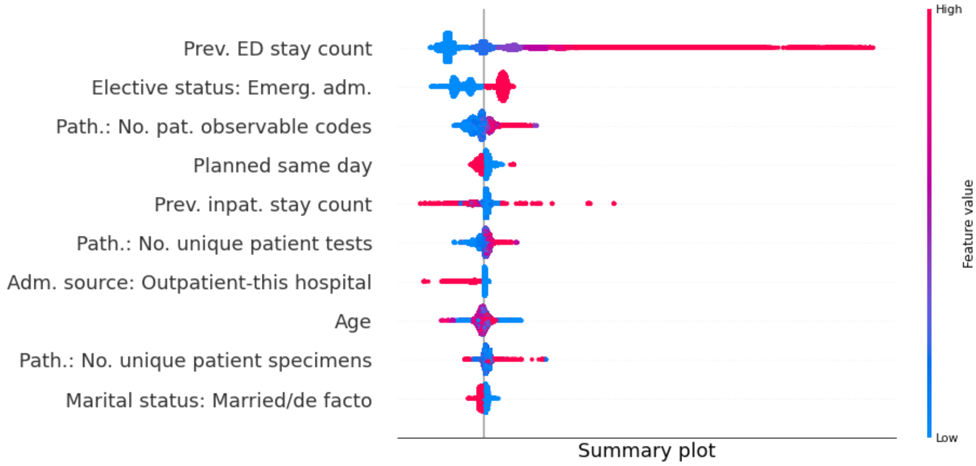

b) **RP30: Public acute hospitals**

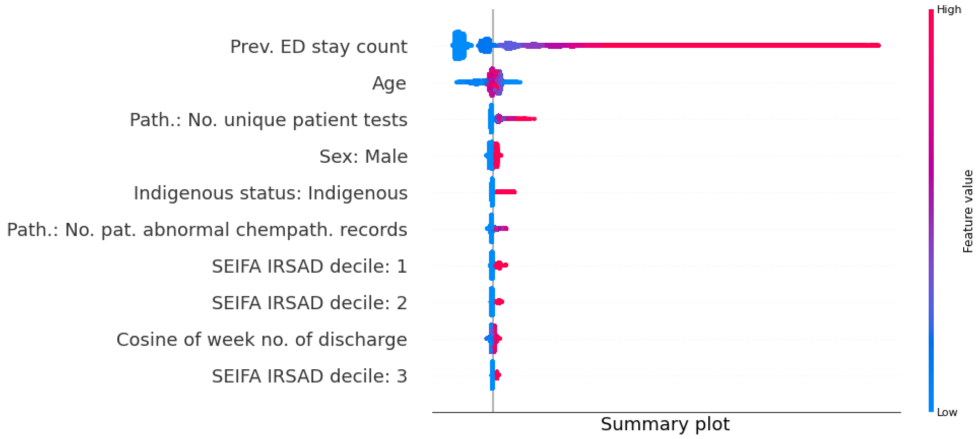

c) **RP30E: Public acute hospitals**

Supplementary Figure S4 Public acute hospitals: Summary plot of Shapley values computed for each patient individually in the test partition. Features are sorted top-down based on their global contribution. The distance of a dot representing a sample from the vertical line indicates its contribution. The colour of a dot indicates feature value for that sample. Blue and pink colour represent extreme values of the feature. Shapley values on the right side of vertical axes 'push' predictions towards the class 1 and those on the left side towards the class 0.

Appendix I Calibration plots for Expert models

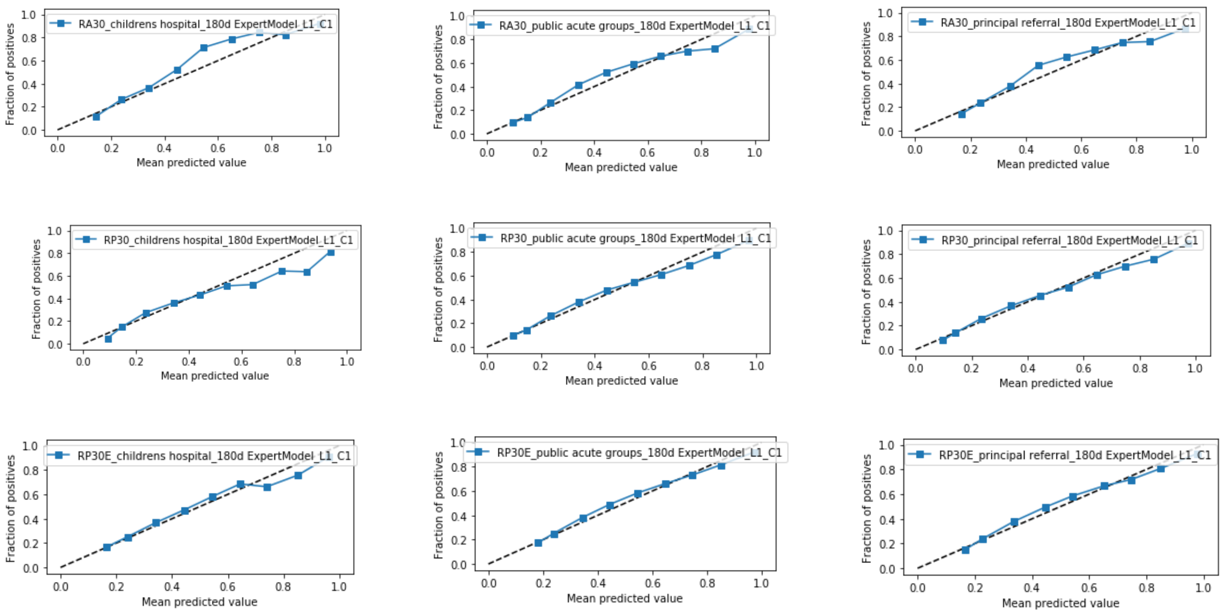

Supplementary Figure S4 Calibration plots obtained for Expert models and logistic regression across the cohorts and prediction outcomes.

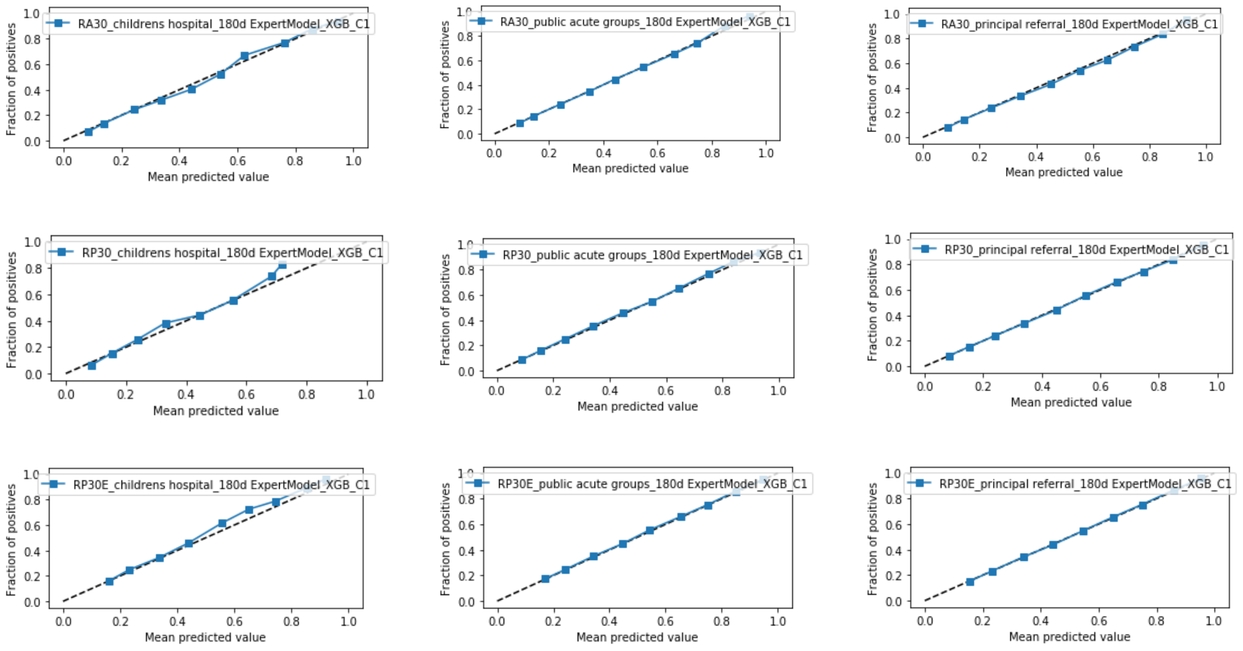

Supplementary Figure S5 Calibration plots obtained for Expert models with XGB across the cohorts and prediction outcomes.

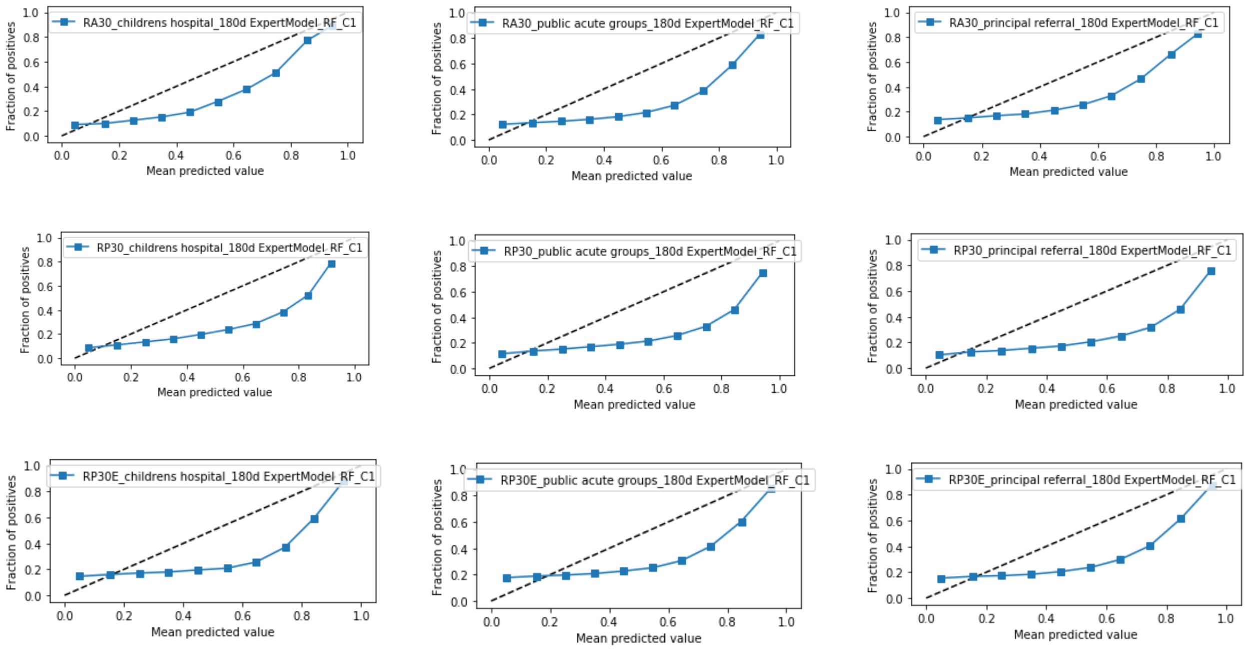

Supplementary Figure S6 Calibration plots obtained for Expert models and RF across the cohorts and prediction outcomes.

Appendix J Expected number of outcomes and actual number of outcomes

Supplementary Table S27 Expected and actual number of outcomes for each cohort computed on the Test data.

Outcome	RA30			RP30			RP30E		
Cohort	Children's hospital	Principal referral	Public acute	Children's hospital	Principal referral	Public acute	Children's hospital	Principal referral	Public acute
Expected*	8162.5	92725.9	95820.7	6539.7	97250.0	121477.9	10063.8	99217.8	196455.6
Actual	7980	92363	96124	6662	98277	124667	10312	98895	199607

*Note: Expected number is the sum of predicted probabilities of the outcome over all stays in the cohort. Actual number is the count of stays in the cohort with a qualifying readmission/re presentation.
